# Supplementary figures and images for: Selective inhibition of mitochondrial Kv1.3 prevents and alleviates multiple sclerosis in vivo
Source: EMBO Mol Med. 2025 Sep 29;17(11):2901–31. doi: 10.1038/s44321-025-00307-2 (PMC12603337; doi:10.1038/s44321-025-00307-2)

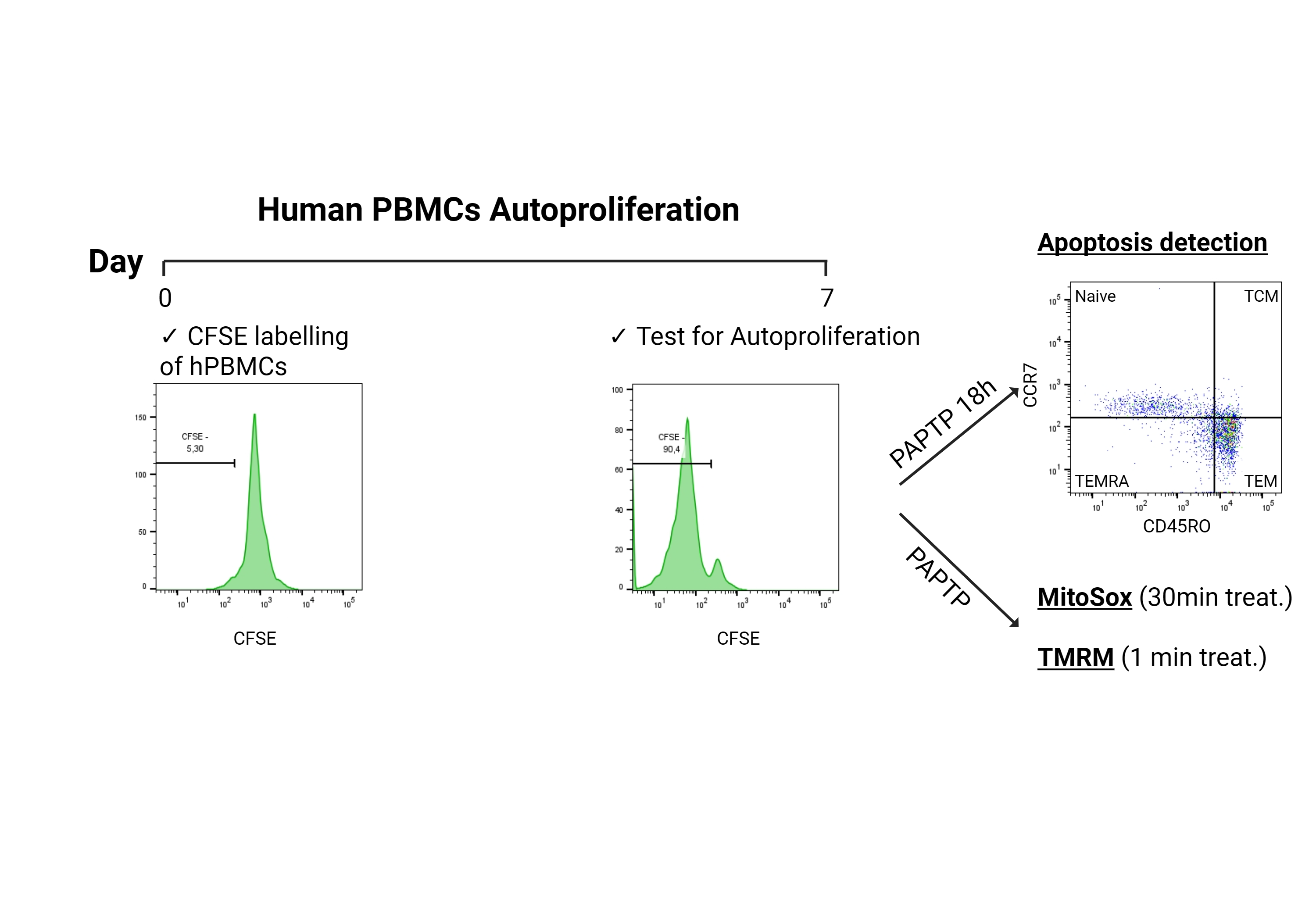

Supplement: Supplementary file 2 — Source data Fig. 1 [file 44321_2025_307_MOESM2_ESM.zip › SourcedataFigure 1/1A/PBMCs autoproliferation.png]

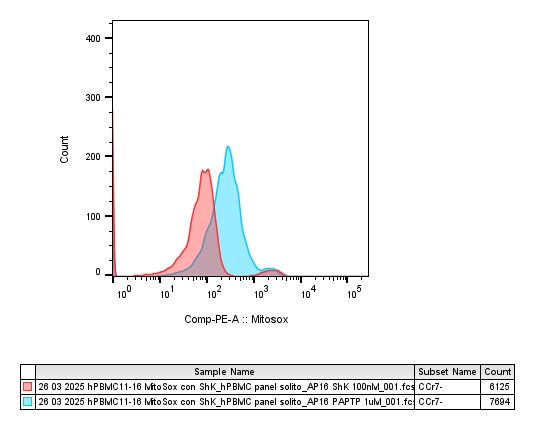

Supplement: Supplementary file 2 — Source data Fig. 1 [file 44321_2025_307_MOESM2_ESM.zip › SourcedataFigure 1/1E/AP16 ShK vs PAPTP.jpg]

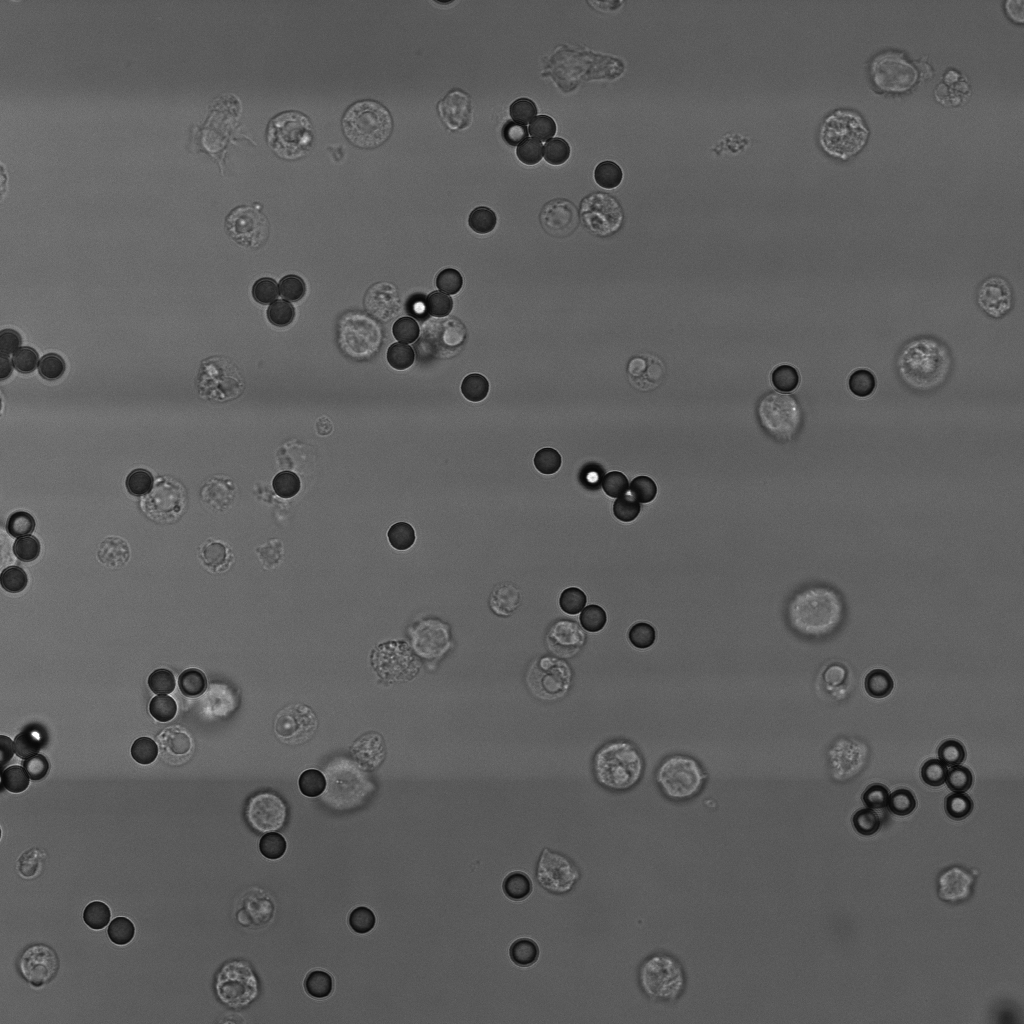

Supplement: Supplementary file 4 — Source data Fig. 3 [file 44321_2025_307_MOESM4_ESM.zip › Source dataFigure 3/3A/PAPTP-Fluor Brightfield.tif]

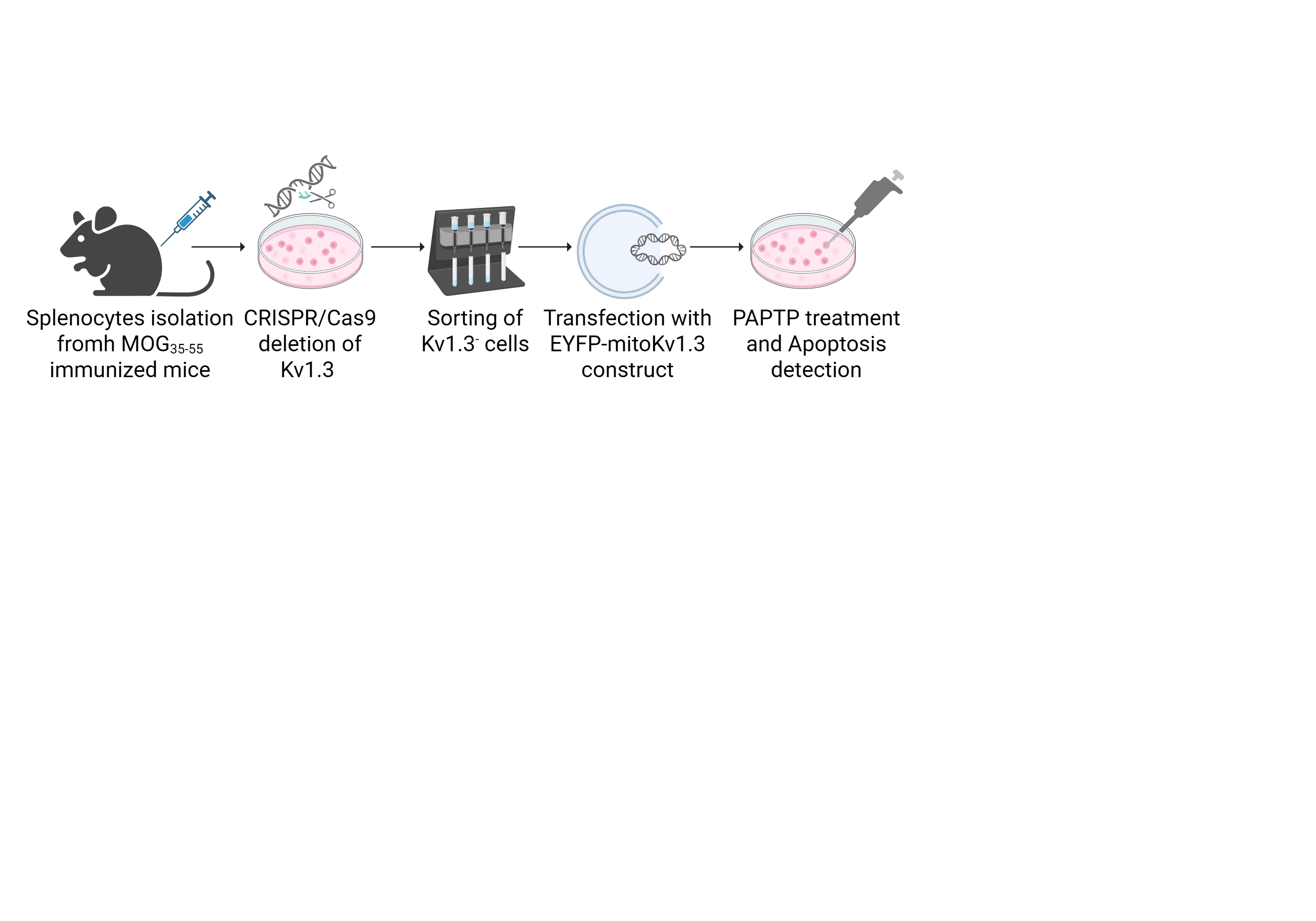

Supplement: Supplementary file 4 — Source data Fig. 3 [file 44321_2025_307_MOESM4_ESM.zip › Source dataFigure 3/3B/CRISPR.png]

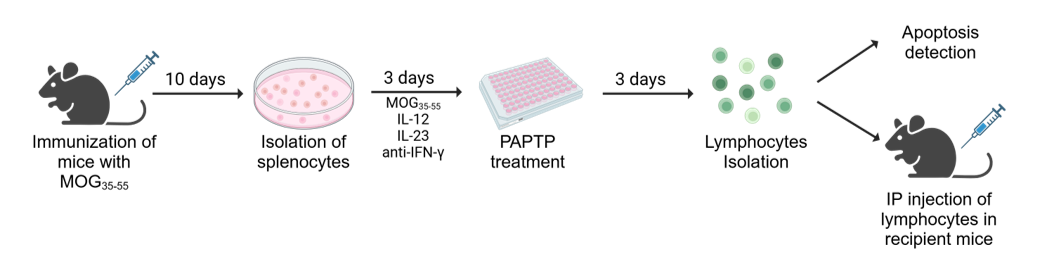

Supplement: Supplementary file 4 — Source data Fig. 3 [file 44321_2025_307_MOESM4_ESM.zip › Source dataFigure 3/3D/Scheme Adoptive Transfer.png]

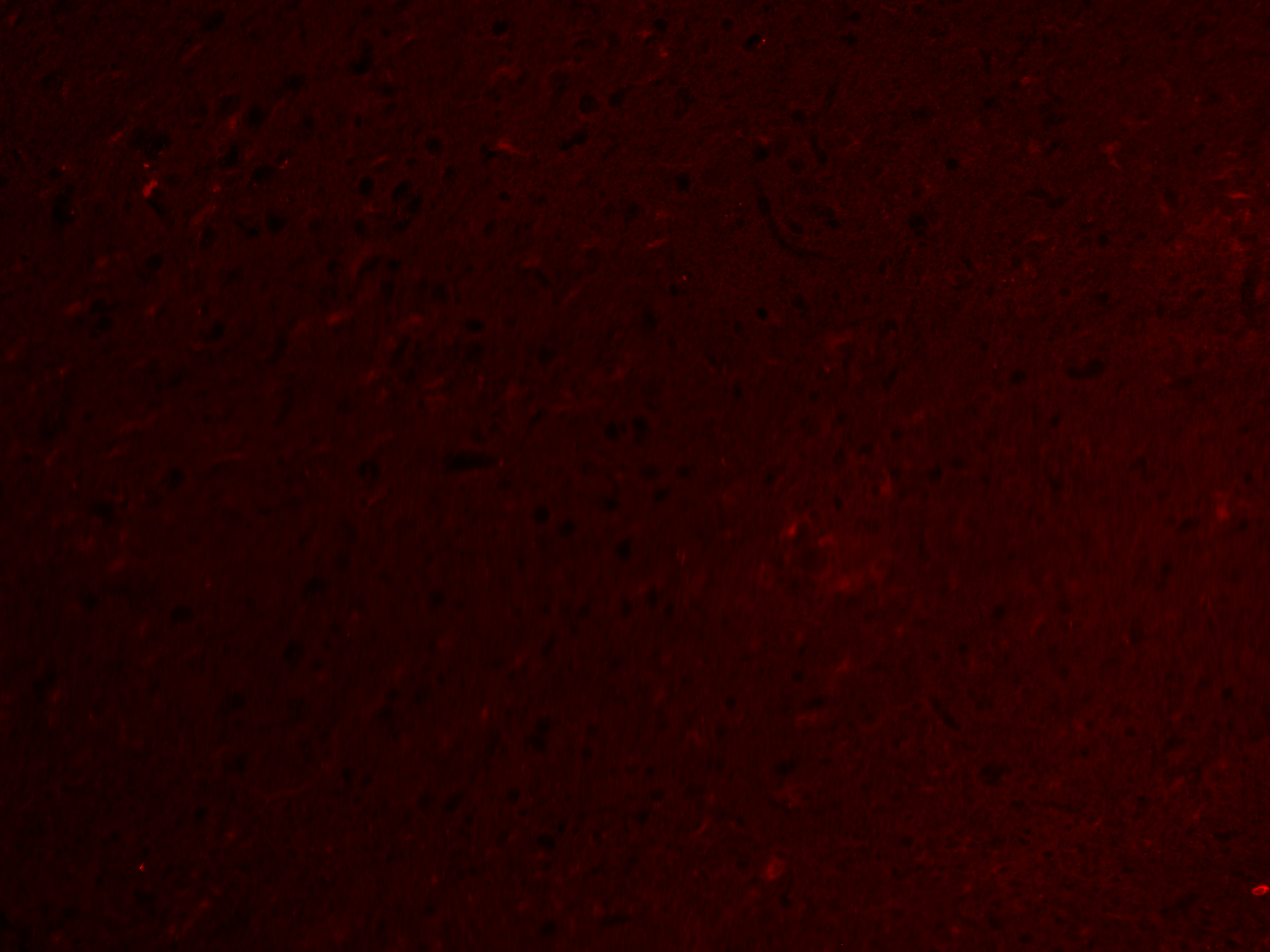

Supplement: Supplementary file 4 — Source data Fig. 3 [file 44321_2025_307_MOESM4_ESM.zip › Source dataFigure 3/3J/1uM PAPTP.tif]

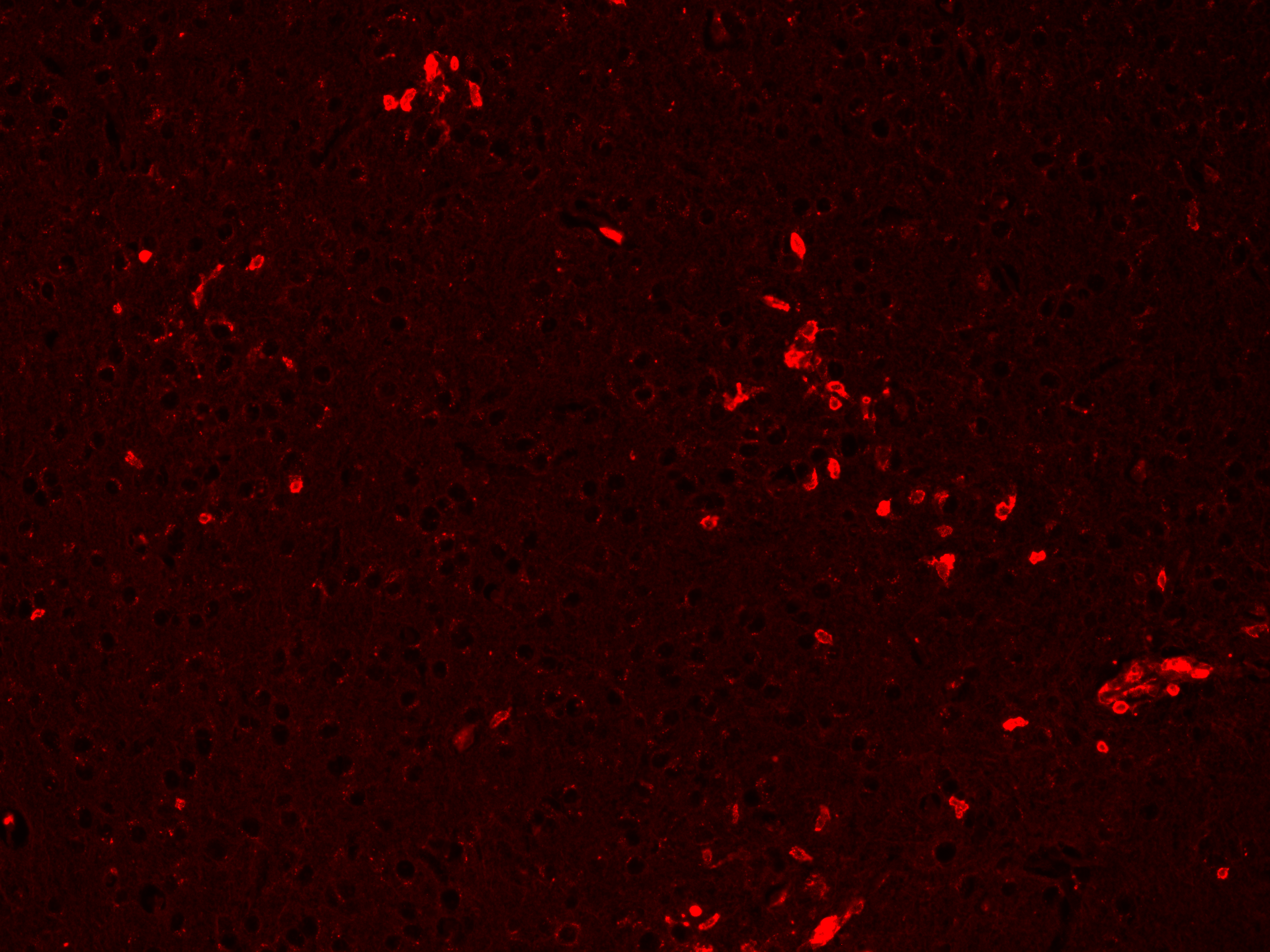

Supplement: Supplementary file 4 — Source data Fig. 3 [file 44321_2025_307_MOESM4_ESM.zip › Source dataFigure 3/3J/Untreated.tif]

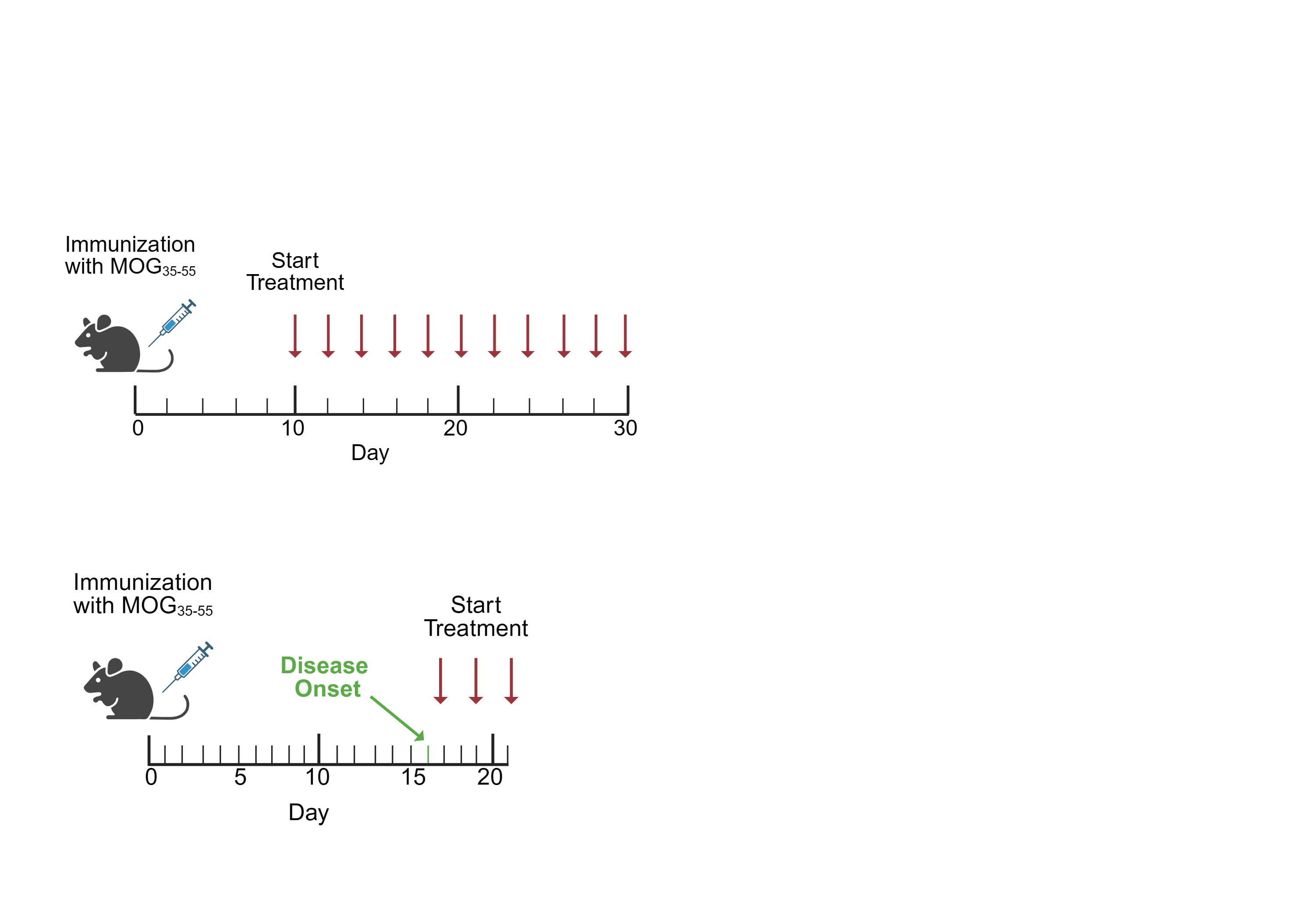

Supplement: Supplementary file 5 — Source data Fig. 4 [file 44321_2025_307_MOESM5_ESM.zip › Source dataFigure 4/4A/EAE treatm scheme (1).png]

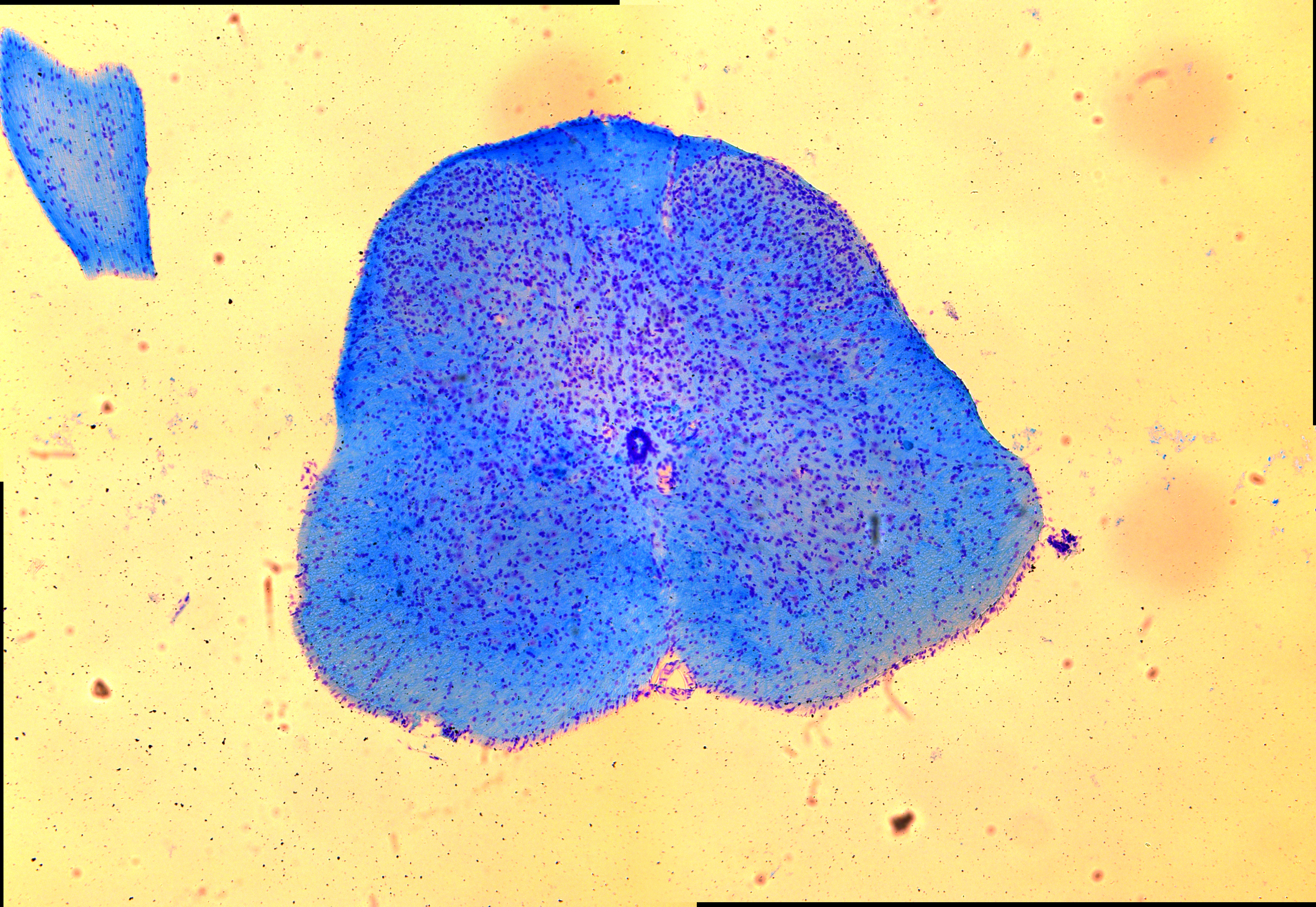

Supplement: Supplementary file 5 — Source data Fig. 4 [file 44321_2025_307_MOESM5_ESM.zip › Source dataFigure 4/4C/CTRL.tif]

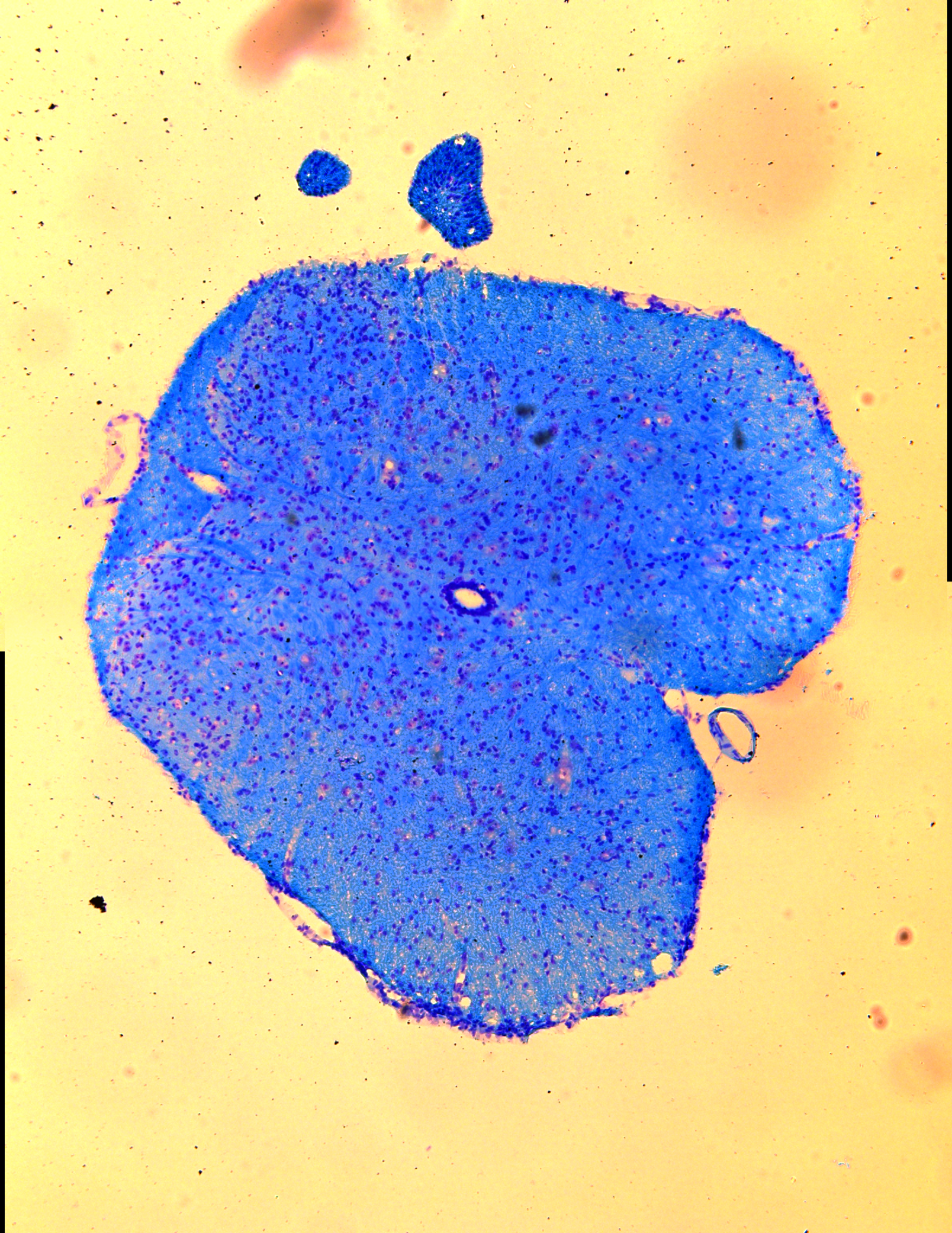

Supplement: Supplementary file 5 — Source data Fig. 4 [file 44321_2025_307_MOESM5_ESM.zip › Source dataFigure 4/4C/EAE+PAPTP.tif]

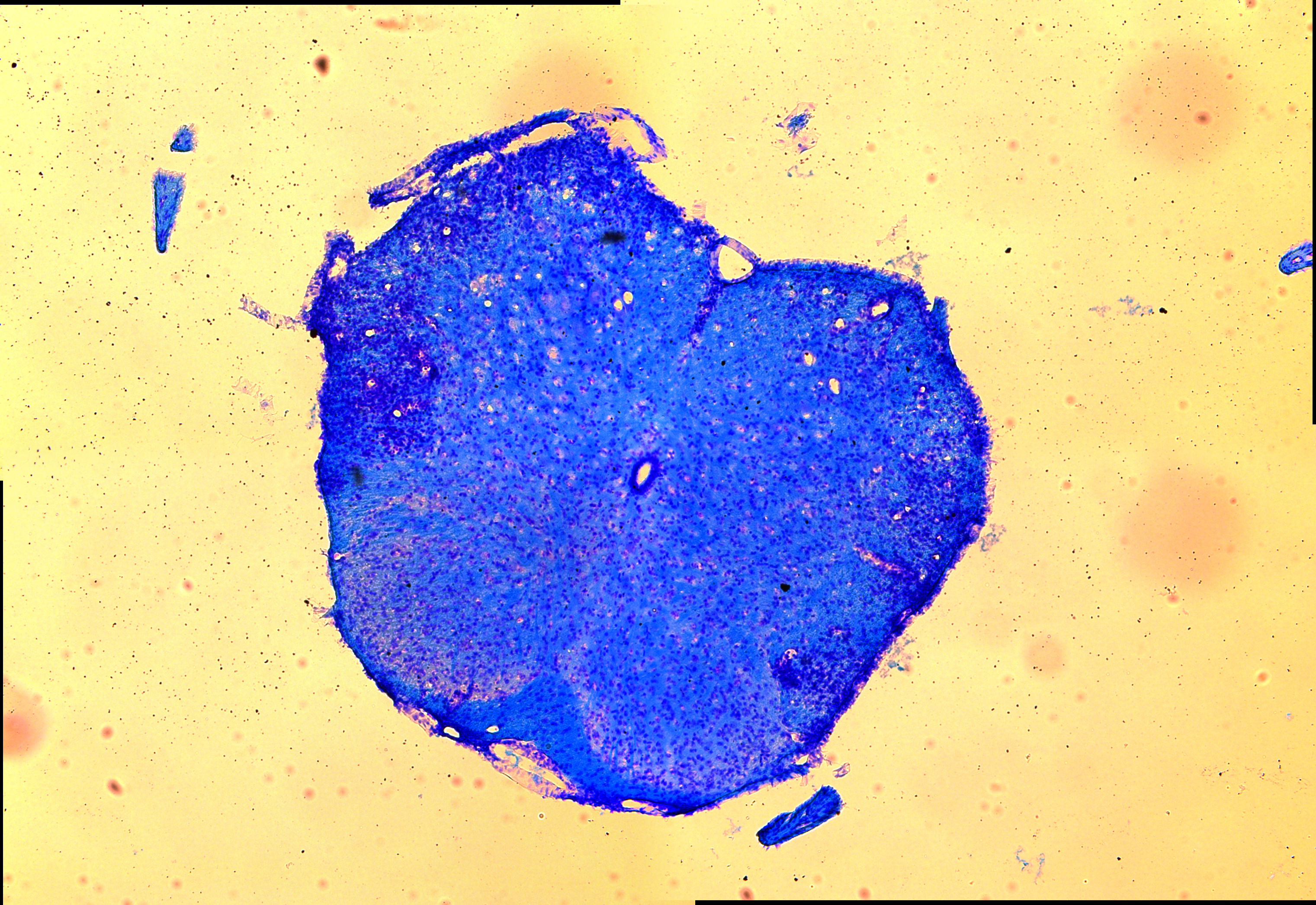

Supplement: Supplementary file 5 — Source data Fig. 4 [file 44321_2025_307_MOESM5_ESM.zip › Source dataFigure 4/4C/EAE.tif]

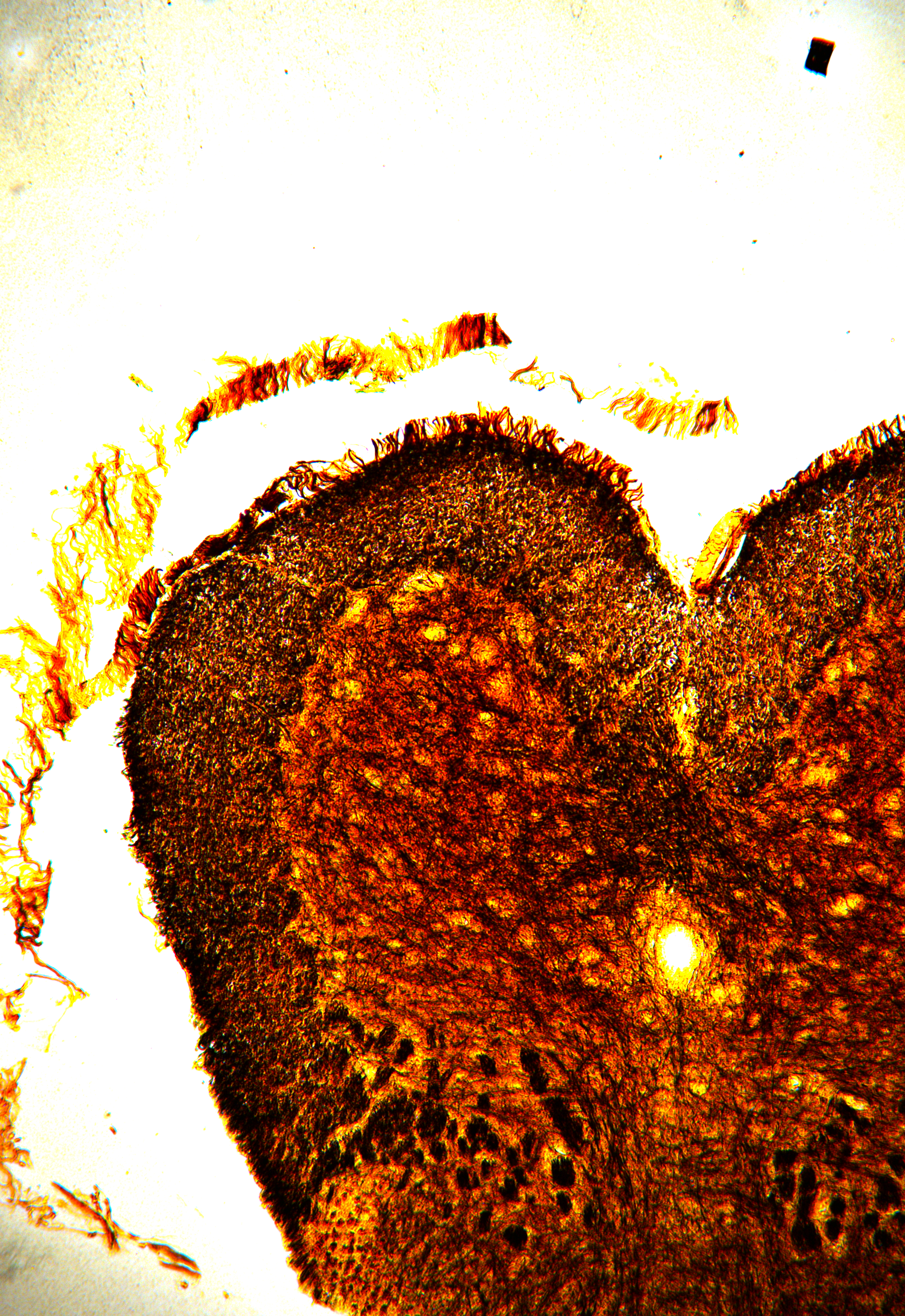

Supplement: Supplementary file 5 — Source data Fig. 4 [file 44321_2025_307_MOESM5_ESM.zip › Source dataFigure 4/4D/Ctrl ZOOM.tif]

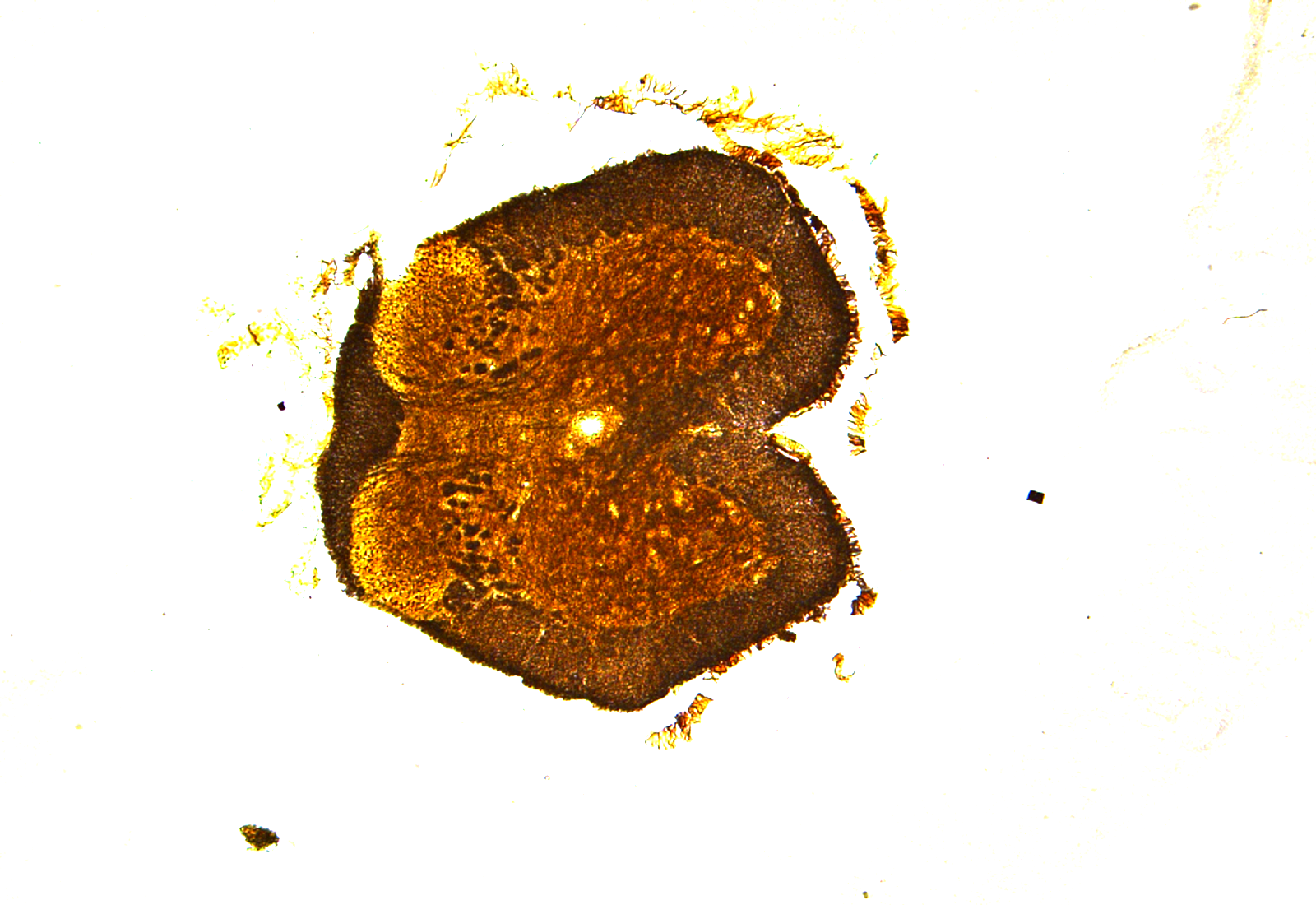

Supplement: Supplementary file 5 — Source data Fig. 4 [file 44321_2025_307_MOESM5_ESM.zip › Source dataFigure 4/4D/CTRL.tif]

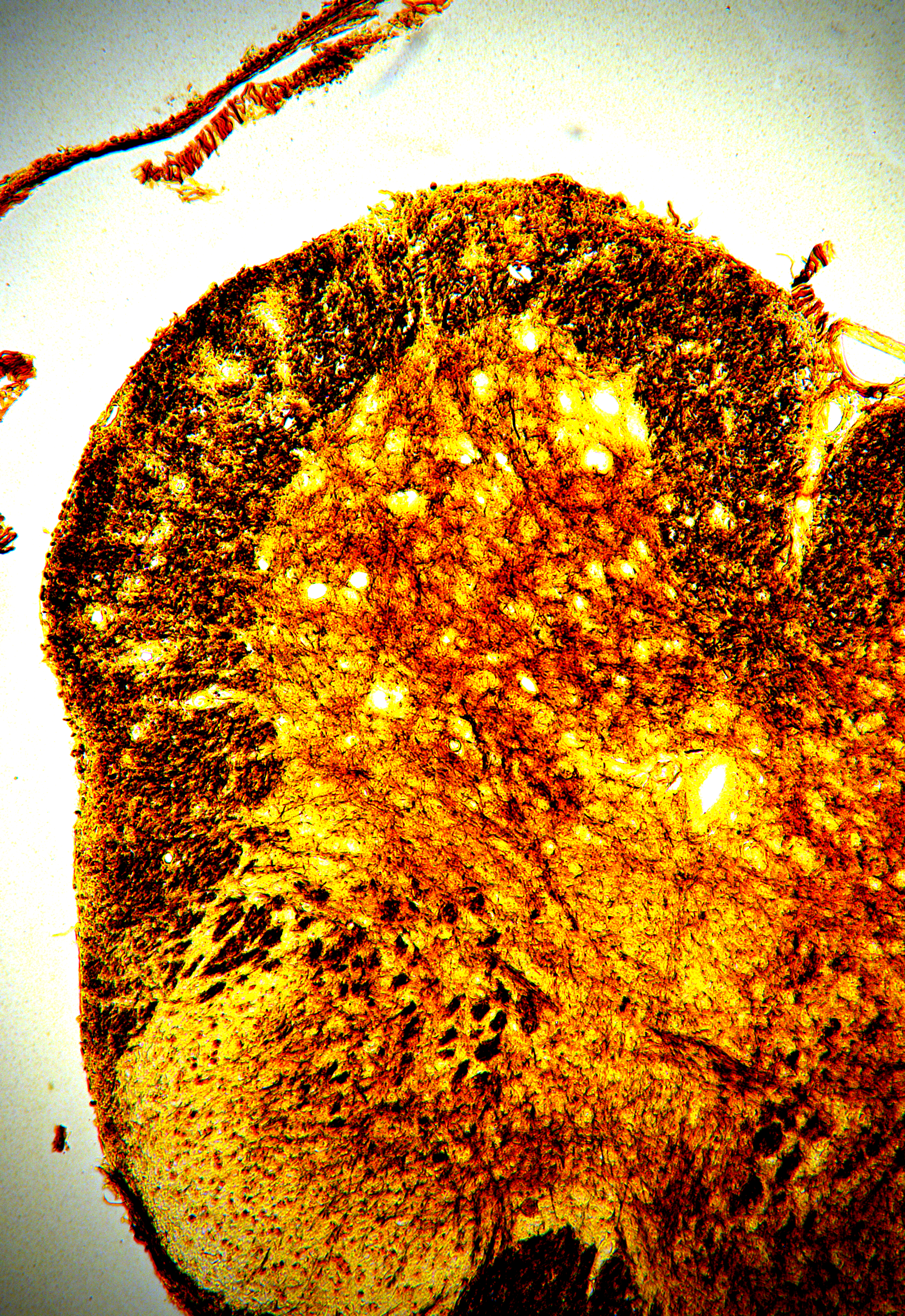

Supplement: Supplementary file 5 — Source data Fig. 4 [file 44321_2025_307_MOESM5_ESM.zip › Source dataFigure 4/4D/EAE Zoom.tif]

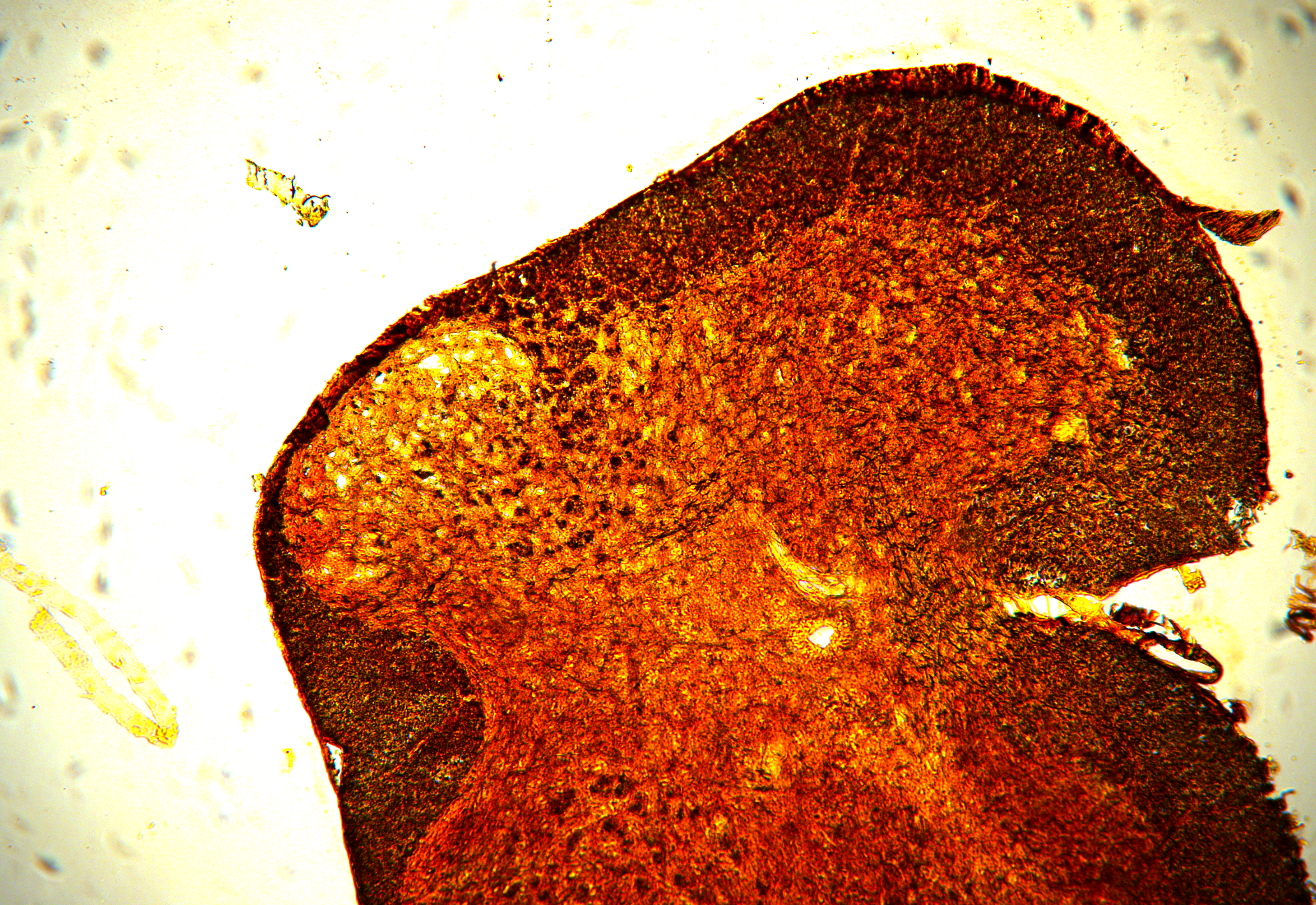

Supplement: Supplementary file 5 — Source data Fig. 4 [file 44321_2025_307_MOESM5_ESM.zip › Source dataFigure 4/4D/EAE+PAPTP Zoom.tif]

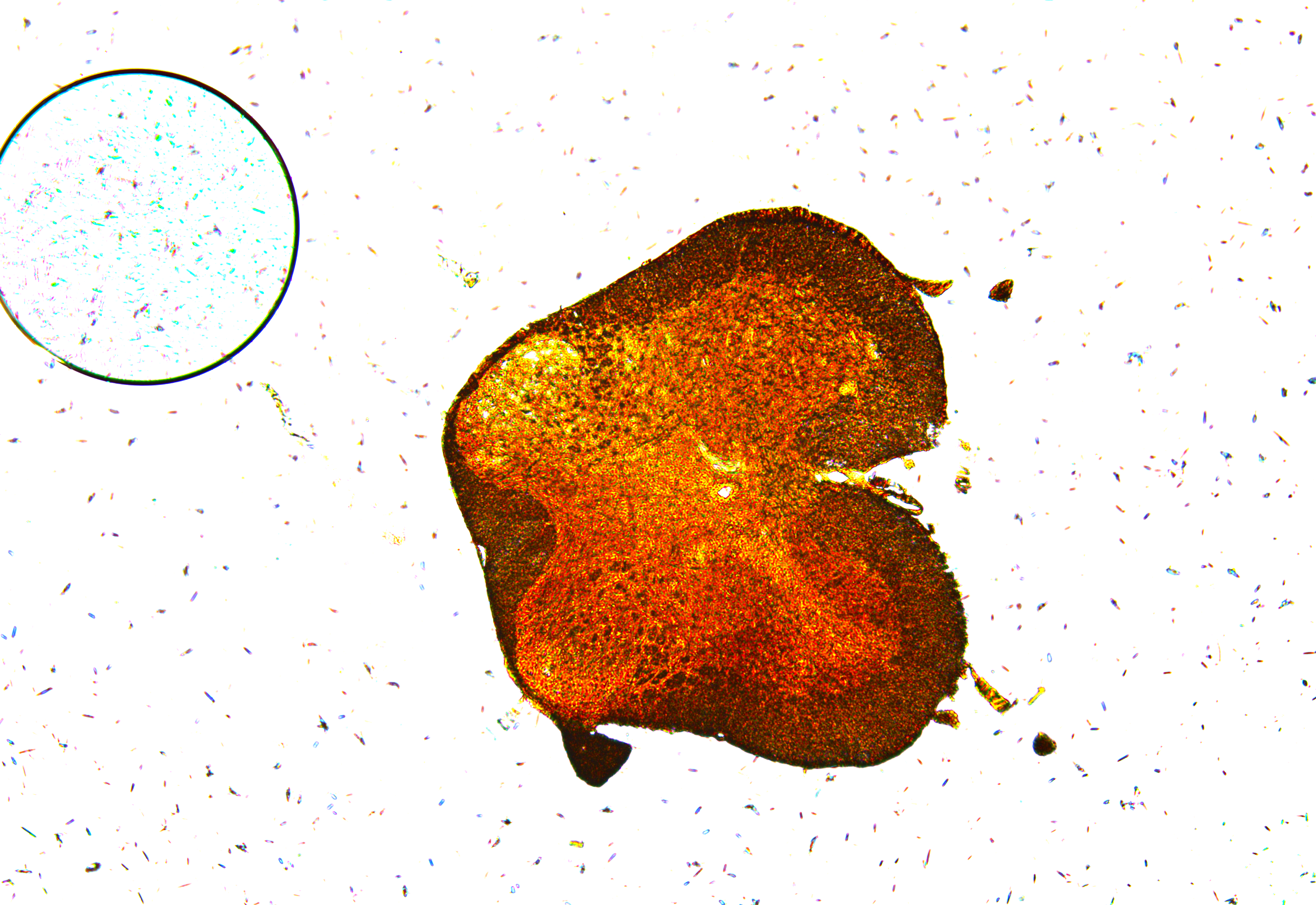

Supplement: Supplementary file 5 — Source data Fig. 4 [file 44321_2025_307_MOESM5_ESM.zip › Source dataFigure 4/4D/EAE+PAPTP.tif]

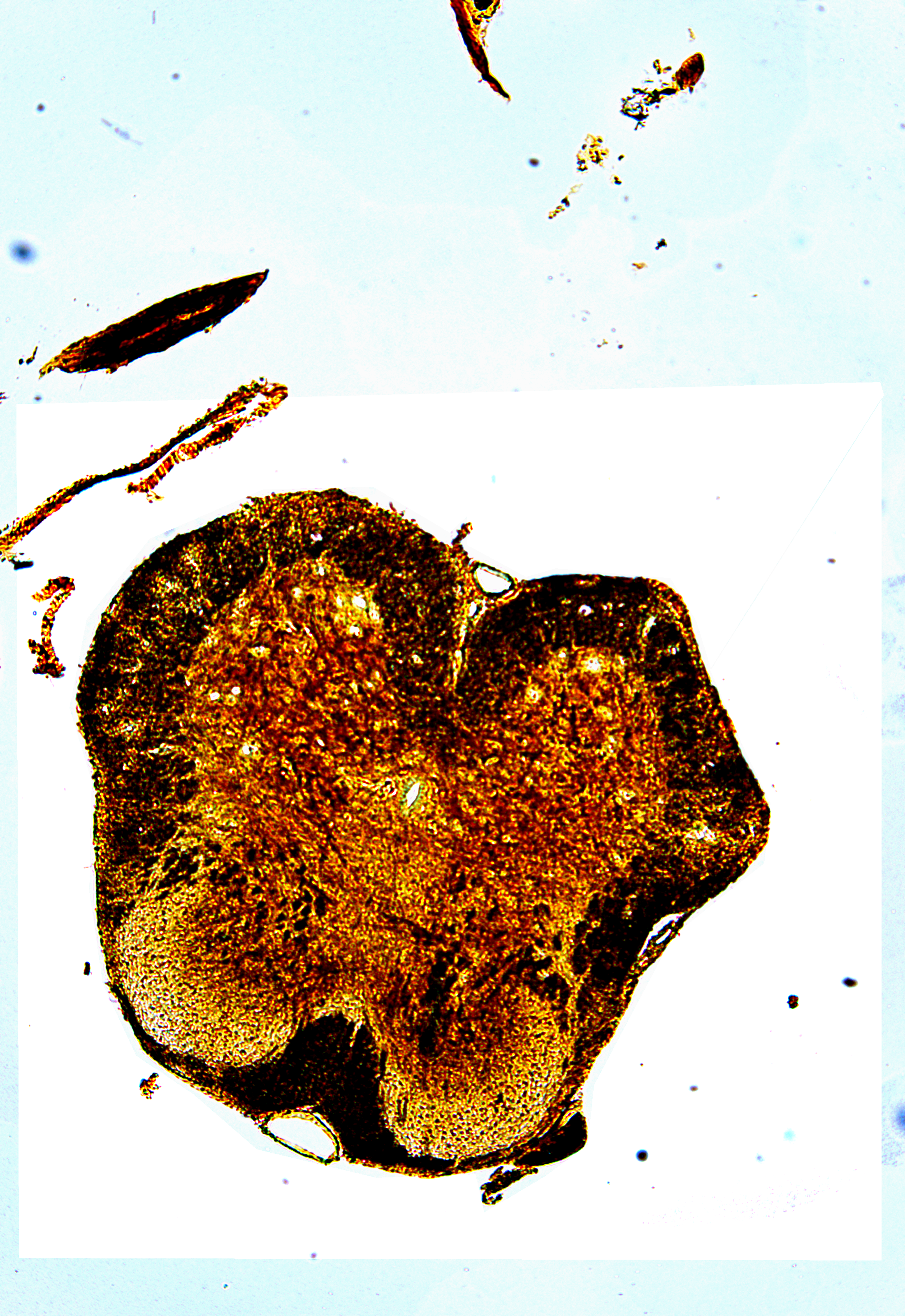

Supplement: Supplementary file 5 — Source data Fig. 4 [file 44321_2025_307_MOESM5_ESM.zip › Source dataFigure 4/4D/EAE.tif]

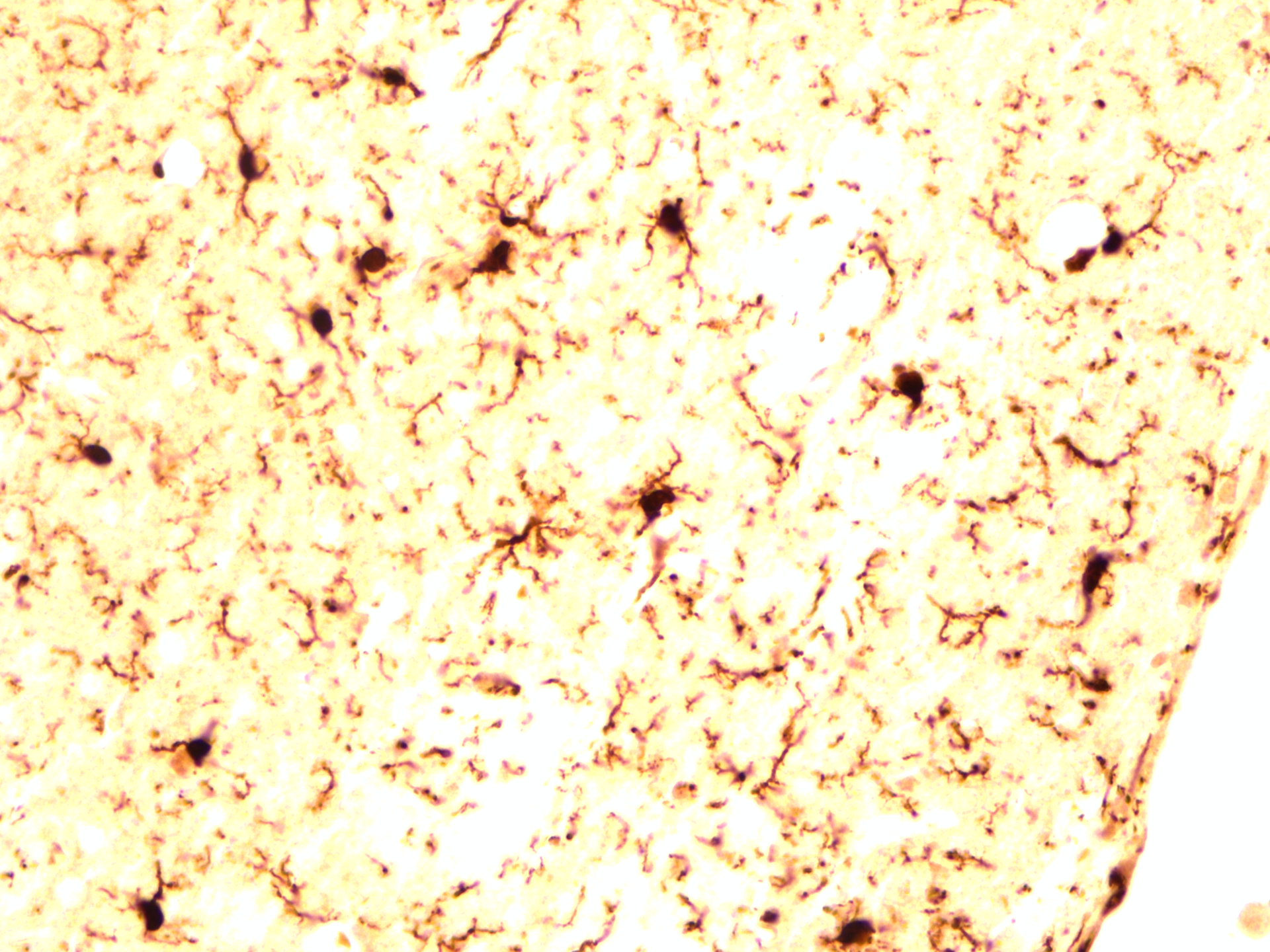

Supplement: Supplementary file 7 — Source data Fig. 5 [file 44321_2025_307_MOESM7_ESM.zip › Source dataFigure 5/5K/ctrl 5-2 40x 6_ch00.tif]

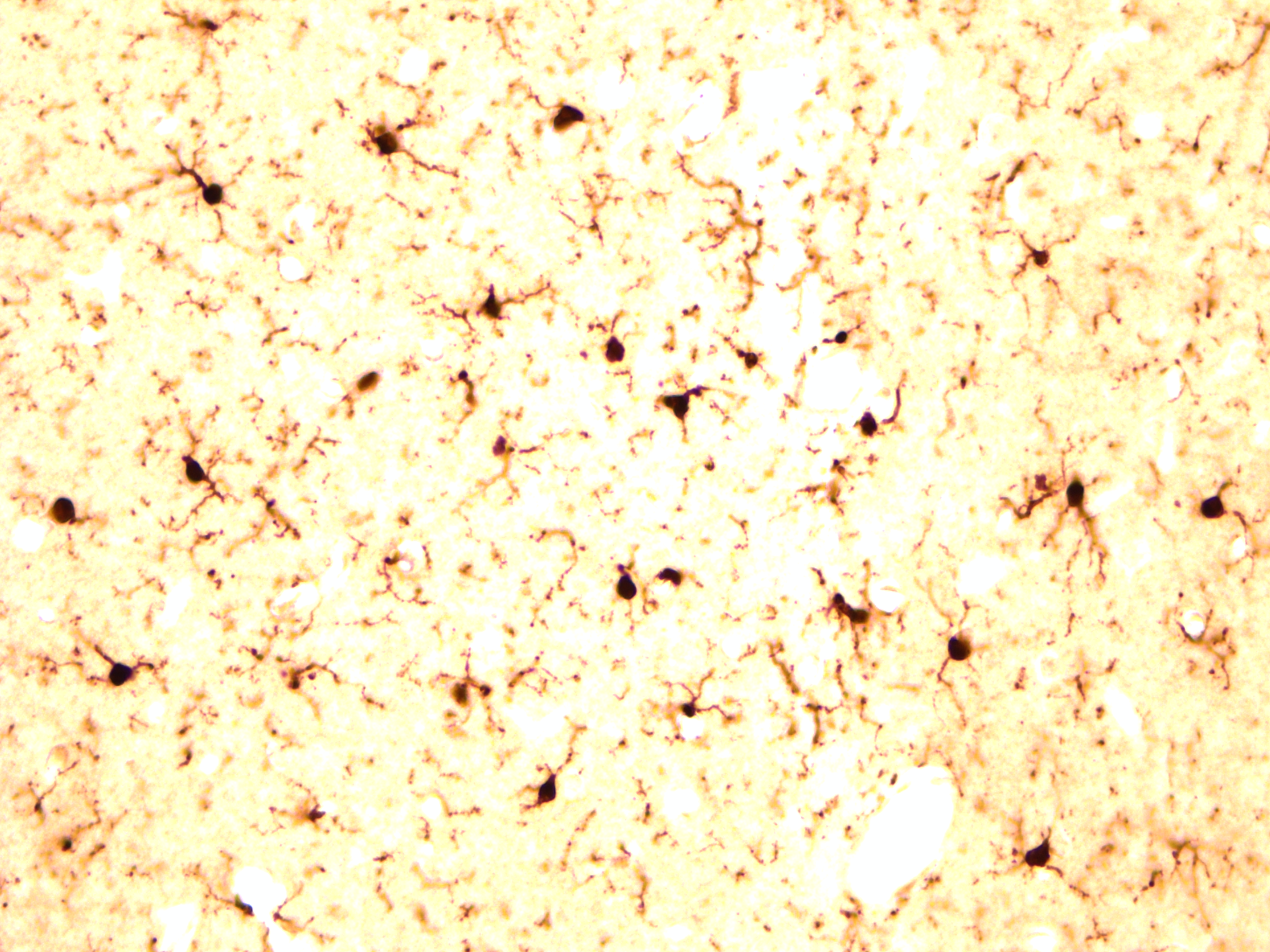

Supplement: Supplementary file 7 — Source data Fig. 5 [file 44321_2025_307_MOESM7_ESM.zip › Source dataFigure 5/5K/EAE 2-3 40x 2_ch00.tif]

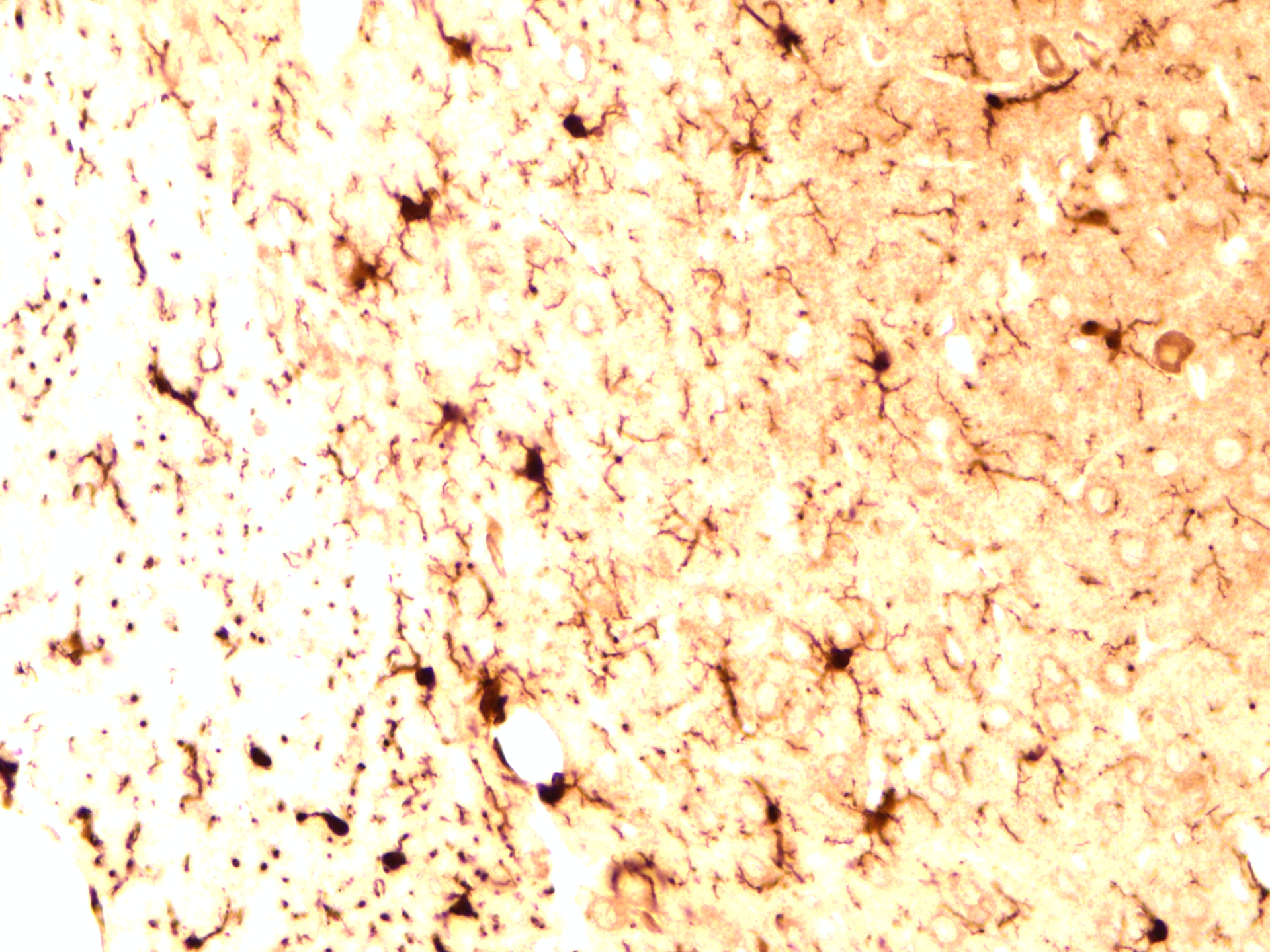

Supplement: Supplementary file 7 — Source data Fig. 5 [file 44321_2025_307_MOESM7_ESM.zip › Source dataFigure 5/5K/EAE PAPTP 3-4 40x _ch00.tif]
