# Supplementary material for: Selective inhibition of mitochondrial Kv1.3 prevents and alleviates multiple sclerosis in vivo
Source: EMBO Mol Med. 2025 Sep 29;17(11):2901–31. doi: 10.1038/s44321-025-00307-2 (PMC12603337; doi:10.1038/s44321-025-00307-2)
Supplement: Supplementary file 11 — Expanded View Figures [file 44321_2025_307_MOESM11_ESM.pdf]

Expanded View Figures

Figure EV1. Analysis of the effects of PAPTP on PBMCs from MS patients.

(**A**) Representative quantitative results of the Mean Fluorescence Intensity of MitoSox in both $CD4^+$ $CD25^+$ $CCR7^+$ (Naive T cells + T_{CM}s) and $CD4^+$ $CD25^+$ $CCR7^-$ ($T_{EM} + T_{EMRA}$) lymphocytes either untreated or treated with 1 μM PAPTP. (**B**) Representative quantitative results displaying the Mean Fluorescence Intensity of ShK-F6CA in $CD4^+$ $CD25^+$ and $CD4^+$ $CD25^-$ lymphocytes in stained hPBMCs (left) and unstained hPBMCs (right). (**C**) Gating Strategy for Apoptosis detection in Naive T cells, T_{EM}, and T_{CM} cells from hPBMCs. (**D**) Percentage of $CCR7^-$ $CD45RO^+$ T_{EM} cells in hPBMCs from MS patients treated with indicated PAPTP concentrations. Cells were gated within $CD4^+$ $CD25^+$ $CFSE^-$ autoproliferative lymphocytes ($n = 10$ for untreated and 5 μM treated groups; $n = 8$ for 1 μM PAPTP group). (**E**) Normalized apoptotic levels of $CCR7^-$ $CD45RO^+$ T_{EM} cells at specified PAPTP concentrations. For each patient, data were normalized based on the untreated sample ($n = 10$ for untreated and 5 μM treated groups; $n = 8$ for 1 μM PAPTP group). (**F**) Percentage of $CCR7^-$ $CD45RO^-$ T_{EMRA} cells in hPBMCs from MS patients treated with indicated PAPTP concentrations. Cells were gated within $CD4^+$ $CD25^+$ $CFSE^-$ autoproliferative lymphocytes ($n = 9$ for untreated and 5 μM treated groups; $n = 8$ for 1 μM PAPTP group). (**G**) Normalized apoptotic levels of $CCR7^-$ $CD45RO^-$ T_{EMRA} cells at specified PAPTP concentrations. For each patient, data were normalized based on the untreated sample ($n = 10$ for untreated and 5 μM treated groups; $n = 8$ for 1 μM PAPTP group). (**H**) Normalized apoptotic levels of $CCR7^-$ effector cells ($T_{EM} + T_{EMRA}$) at specified PAPTP concentrations. For each patient, data were normalized based on the untreated sample ($n = 10$ for untreated and 5 μM treated groups; $n = 8$ for 1 μM PAPTP group). (**I**) Percentage of $CCR7^+$ $CD45RO^-$ naive T cells in hPBMCs from MS patients treated with indicated PAPTP concentrations. Cells were gated within $CD4^+$ $CD25^+$ $CFSE^-$ autoproliferative lymphocytes ($n = 7$ for untreated and 5 μM PAPTP groups; $n = 5$ for 1 μM PAPTP). (**J**) Normalized apoptotic levels of $CCR7^+$ $CD45RO^-$ naive T cells at specified PAPTP concentrations. For each patient, data were normalized based on the untreated sample ($n = 7$ for untreated and 5 μM PAPTP groups; $n = 5$ for 1 μM PAPTP). (**K**) Percentage of $CCR7^+$ $CD45RO^+$ T_{CM} cells in hPBMCs from MS patients treated with indicated PAPTP concentrations. Cells were gated within $CD4^+$ $CD25^+$ $CFSE^-$ autoproliferative lymphocytes ($n = 8$ for untreated and 5 μM PAPTP groups; $n = 6$ for 1 μM PAPTP). (**L**) Normalized apoptotic levels of $CCR7^+$ $CD45RO^+$ T_{CM} cells at specified PAPTP concentrations. For each patient, data were normalized based on the untreated sample ($n = 8$ for untreated and 5 μM PAPTP groups; $n = 6$ for 1 μM PAPTP). Two outliers were removed from the graph. (**M**) Percentages of apoptotic cells of the indicated $CD4^+$ $CD25^+$ subpopulations obtained from healthy subjects PBMC ($n = 5$ for each indicated condition). (**N**) Gating strategy for the identification of $CCR7^+$ and $CCR7^-$ cells for MitoSOX analysis. Mitosox Mean Fluorescence Intensity was evaluated on $CCR7^+$ and $CCR7^-$ populations. The same gating strategy was used for the analysis of Kv1.3 expression, using ShK-F6CA. The ShK-F6CA Mean Fluorescence Intensity of $CD4^+$ $CD25^+$ ShK-F6CA and $CD4^+$ $CD25^-$ cells was evaluated. (**D–M**) Data represent average ± SEM with superimposed individual data points for each patient. Each data point represents hPBMCs derived from a distinct patient. (**D**) *p*-values of one-way ANOVA test. (**E**, **G**, **H**, **J**, **L**) *p*-values of Wilcoxon test. (**N**) *p*-value in Friedman test. Where values are not indicated, no significant difference was observed.

 © The Author(s)

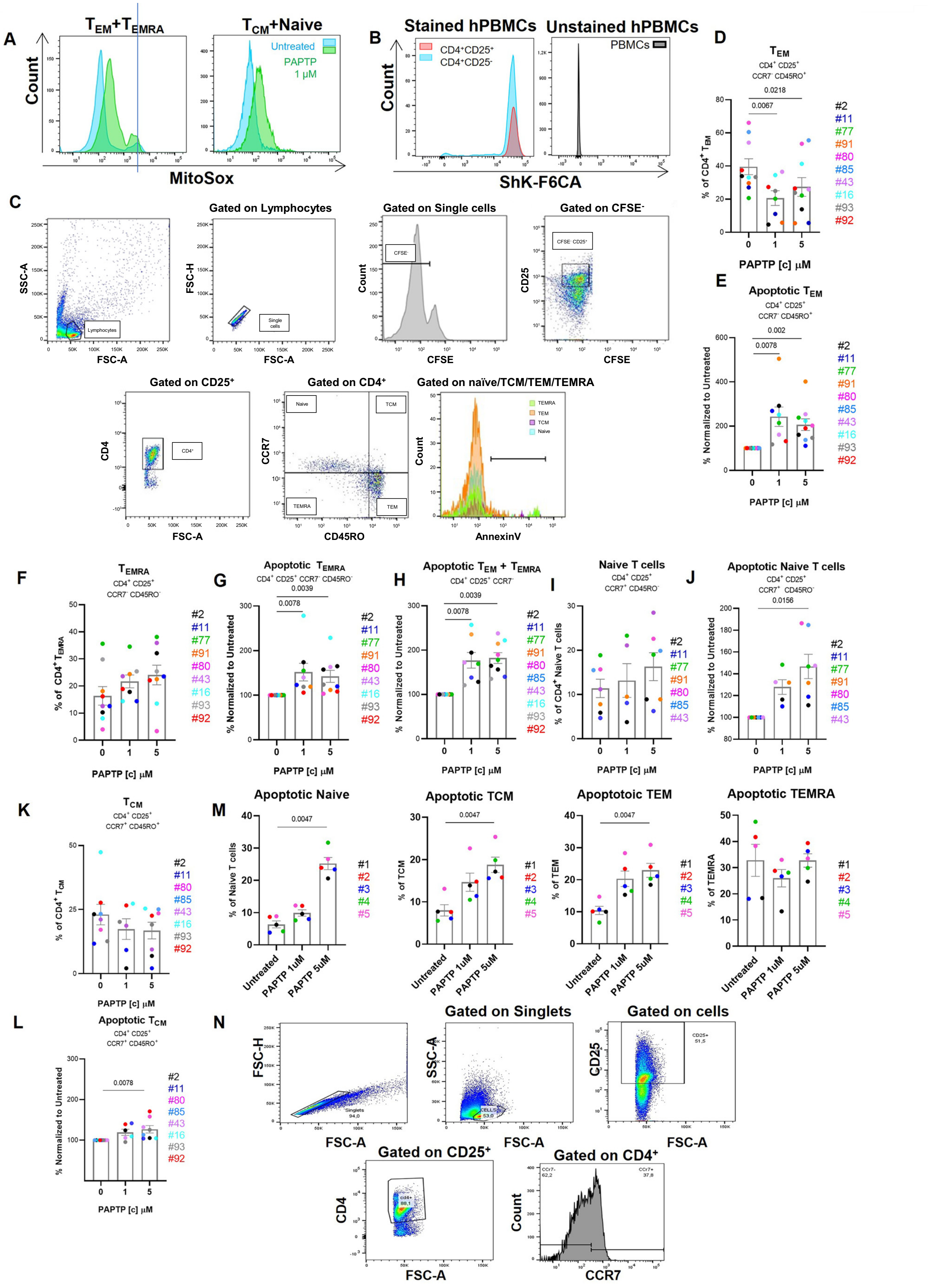
A
TEM+TEMRA
TCM+Naive
Count
MitoSox
Untreated
PAPTP 1 µM
B
Stained hPBMCs
Unstained hPBMCs
CD4+CD25+
CD4+CD25-
PBMCs
ShK-F6CA
C
Gated on Lymphocytes
Gated on Single cells
Gated on CFSE-
Gated on CD25+
Gated on CD4+
Gated on naïve/TCM/TEM/TEMRA
D
E
Apoptotic TEM
F
G
Apoptotic TEMRA
H
Apoptotic TEM + TEMRA
I
Naive T cells
J
Apoptotic Naive T cells
K
M
Apoptotic Naive
Apoptotic TCM
Apoptotoic TEM
Apoptotic TEMRA
L
Apoptotic TCM
N
Gated on Singlets
Gated on cells
Gated on CD25+
Gated on CD4+
PAPTP [c] µM

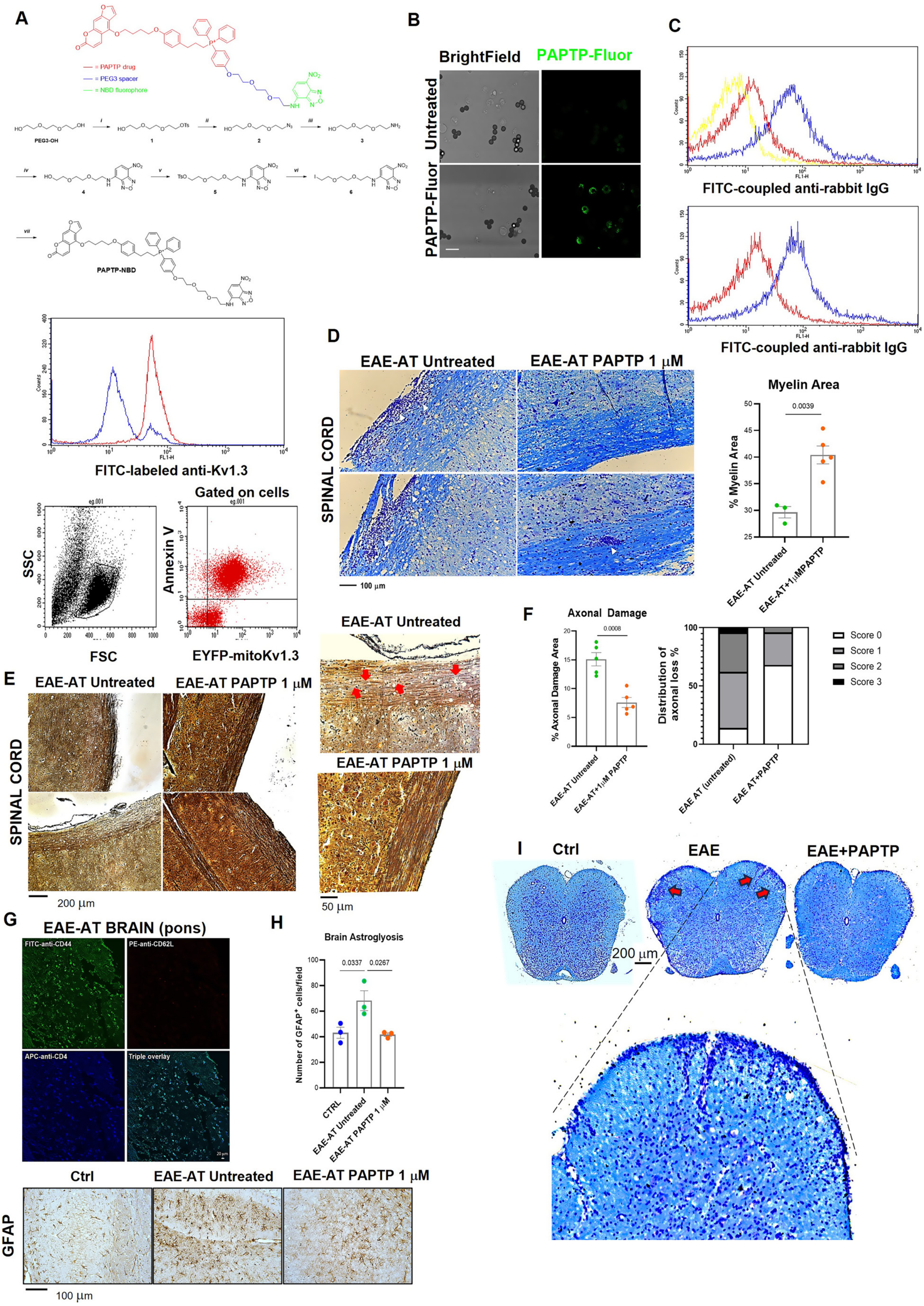
A
= PAPTP drug
= PEG3 spacer
= NBD fluorophore
PEG3-OH
PAPTP-NBD
FITC-labeled anti-Kv1.3
Gated on cells
SSC
FSC
Annexin V
EYFP-mitoKv1.3
B
BrightField
PAPTP-Fluor
Untreated
PAPTP-Fluor
C
FITC-coupled anti-rabbit IgG
FITC-coupled anti-rabbit IgG
D
EAE-AT Untreated
EAE-AT PAPTP 1 μM
SPINAL CORD
100 μm
Myelin Area
0.0039
% Myelin Area
EAE-AT Untreated
EAE-AT+1μMPAPTP
E
EAE-AT Untreated
EAE-AT PAPTP 1 μM
SPINAL CORD
200 μm
EAE-AT Untreated
EAE-AT PAPTP 1 μM
50 μm
F
Axonal Damage
0.0008
% Axonal Damage Area
EAE-AT Untreated
EAE-AT+1μM PAPTP
Distribution of axonal loss %
EAE AT (untreated)
EAE AT+PAPTP
Score 0
Score 1
Score 2
Score 3
G
EAE-AT BRAIN (pons)
FITC-anti-CD44
PE-anti-CD62L
APC-anti-CD4
Triple overlay
Ctrl
EAE-AT Untreated
EAE-AT PAPTP 1 μM
GFAP
100 μm
H
Brain Astrogliosis
0.0337
0.0267
Number of GFAP+ cells/field
CTRL
EAE-AT Untreated
EAE-AT PAPTP 1 μM
I
Ctrl
EAE
EAE+PAPTP
200 μm

 © The Author(s)

Figure EV2. PAPTP prevents EAE onset in the Adoptive Transfer Model.

(**A**) Upper panel: Chemical Structure of 7-nitrobenz-2-oxa-1,3-diazole (NBD)-labeled PAPTP (PAPTP-NBD). Lower panel: Synthesis of PAPTP-NBD. Reagents and Conditions (i) TsCl, DMAP, pyridine, DCM, r.t., 18 h; (ii) NaN_3, DMF, 90 °C, 3 h; (iii) Pd/C, H2, MeOH, r.t., 16 h; (iv) NBD-Cl, DIPEA, MeOH, r.t., 16 h; (v) TsCl, DMAP, pyridine, DCM, r.t., 5 h; (vi) NaI, acetone, 40 °C, 16 h; (vii) PAPTP-OH, K_2CO_3, DMF, r.t., 16 h. Synthesis of 2-(2-(2-hydroxyethoxy)ethoxy)ethyl 4-methylbenzenesulfonate (1). To a solution of triethylene glycol (PEG3-OH, 77.28 g, 514.6 mmol, 8.0 equiv) in dichloromethane (DCM, 255 mL) at 0 °C were added 4-dimethylaminopyridine (DMAP, 15.7 g, 128.6 mmol, 2.0 equiv) and pyridine (10.18 g, 128.6 mmol, 2.0 equiv). After 10 min of stirring, p-toluenesulfonyl chloride (TsCl, 12.3 g, 64.3 mmol, 1.0 equiv), previously dissolved in DCM (165 mL), was added dropwise. The reaction mixture was stirred at room temperature for 18 h. The reaction was then quenched by dilution with 300 mL of 1.0 M HCl, the organic layer separated and the aqueous phase was extracted with DCM (2 × 250 mL). The combined organic layers were dried over anhydrous Na_2SO_4, filtered, and concentrated under reduced pressure. The crude product was purified by flash column chromatography on silica gel using a DCM/acetone mixture (8:2) to afford 1 as a pale yellow oil (13.2 g, 43.4 mmol, yield: 67%). 1H NMR (400 MHz, CDCl3) δ 7.80 (d, J = 8.4 Hz, 2H), 7.34 (d, J = 8.4 Hz, 2H), 4.18–4.15 (m, 2H), 3.72–3.69 (m, 4H), 3.61 (s, 4H), 3.58–3.56 (m, 2H), 2.44 (s, 3H).13 C NMR (101 MHz, CDCl3) δ 145.01, 133.07, 129.98, 128.11, 72.59, 70.92, 70.43, 69.29, 68.85, 61.89, 21.78. ESI-MS (ion trap): *m/z* 305 [M + H]+. Synthesis of 2-(2-(2-azidoethoxy)ethoxy)ethan-1-ol (2). To a solution of compound 1 (13.2 g, 43.4 mmol, 1.0 equiv) in anhydrous N,N-dimethylformamide (DMF, 100 mL), sodium azide (NaN_3, 8.5 g, 130.2 mmol, 3.0 equiv) was added. The reaction mixture was stirred at 90 °C for 3 h until thin-layer chromatography (TLC) analysis (EtOAc/PE, 6:4) indicated complete consumption of the starting material. The reaction mixture was then diluted with ethyl acetate (EtOAc, 300 mL) and washed with brine/water 1:1 (5 × 100 mL). The organic layer was dried, and the solvent was removed under reduced pressure. The flask was left under high vacuum overnight to remove residual DMF, yielding compound 2 as a pale yellow oil (6.8 g, 38.7 mmol, yield: 89%). 1H NMR (400 MHz, CDCl3) δ 3.73–3.71 (m, 2H), 3.68–3.64 (m, 6H), 3.61–3.59 (m, 2H), 3.38 (t, J = 5.0 Hz, 2H), 2.45 (s, 1H).13 C NMR (101 MHz, CDCl3) δ 72.59, 70.74, 70.48, 70.13, 61.84, 50.74. ESI-MS (ion trap): *m/z* 176 [M + H]+. Synthesis of 2-(2-(2-aminoethoxy)ethoxy)ethan-1-ol (3). Palladium on carbon (Pd/C, 10% w/w, 0.70 g) was suspended in methanol (30 mL) in a round-bottom flask under nitrogen atmosphere. Compound 2 (6.8 g, 38.7 mmol, 1.0 equiv), previously dissolved in methanol (10 mL), was added to the suspension. The reaction atmosphere was then replaced with hydrogen, and the mixture was stirred at room temperature for 16 h, until thin-layer chromatography (TLC) analysis (DCM/acetone, 8:2) confirmed complete consumption of the starting material. Hydrogen was removed by nitrogen stream and the reaction mixture was filtered through a celite pad to remove the catalyst. The solvent was evaporated under reduced pressure and the crude product was purified by flash column chromatography on silica gel using a DCM/MeOH/NH3(aq) (87:12:1) as the eluent, affording compound 3 as a colorless oil (2.6 g, 17.4 mmol, yield: 45%). 1H NMR (400 MHz, CDCl3) δ 3.73–3.70 (m, 2H), 3.68–3.62 (m, 4H), 3.61–3.58 (m, 2H), 3.56–3.53 (m, 2H), 2.88 (t, J = 5.1 Hz, 2H), 2.55 (s, 3H).13 C NMR (101 MHz, CDCl3) δ 72.81, 72.79, 70.47, 70.24, 61.63, 41.53. ESI-MS (ion trap): *m/z* 150 [M + H]+. Synthesis of 2-(2-(2-((7-nitrobenzo[c][1,2,5]oxadiazol-4-yl)amino)ethoxy)ethoxy)ethan-1-ol (4). To a solution of NBD-Cl (1.0 g, 5.0 mmol, 1.0 equiv) in methanol (25 mL) at 0 °C were added N,N-diisopropylethylamine (DIPEA, 2.6 g, 20 mmol, 4.0 equiv) and compound 3 (0.82 g, 5.5 mmol, 1.1 equiv). The reaction mixture was allowed to warm to room temperature and stirred for 16 h. The mixture was then diluted with ethyl acetate (150 mL) and washed with saturated NH4Cl solution (3 × 50 mL). The organic layers were combined, dried, and concentrated under reduced pressure. The crude product was purified by flash column chromatography on silica gel using an EtOAc/MeOH mixture (99:1) as the eluent, affording compound 4 as a brown powder (1.0 g, 3.2 mmol, yield: 64%). 1H NMR (400 MHz, MeOD) δ 8.51 (d, J = 8.9 Hz, 1H), 6.43 (d, J = 8.9 Hz, 1H), 3.84–3.80 (m, 2H), 3.76 (s, 2H, broad signal), 3.71–3.66 (m, 2H), 3.66–3.62 (m, 4H), 3.56–3.53 (m, 2H).13 C NMR (101 MHz, DMSO) δ 154.82, 153.90, 153.60, 147.35, 130.29, 109.01, 81.83, 79.36, 79.22, 77.44, 69.66, 52.88. ESI-MS (ion trap): *m/z* 313 [M + H]+. Synthesis of 2-(2-(2-((7-nitrobenzo[c][1,2,5]oxadiazol-4-yl)amino)ethoxy)ethoxy)ethyl 4-methylbenzenesulfonate (5). To a solution of compound 4 (100 mg, 0.32 mmol, 1.0 equiv) in dichloromethane (DCM, 1.5 mL) at 0 °C were added pyridine (76 mg, 0.96 mmol, 3.0 equiv), 4-dimethylaminopyridine (DMAP, 78 mg, 0.64 mmol, 2.0 equiv), and p-toluenesulfonyl chloride (TsCl, 122 mg, 0.64 mmol, 2.0 equiv). The reaction mixture was stirred at room temperature for 5 h. The mixture was then diluted with brine (50 mL) and extracted with DCM (3 × 50 mL). The combined organic layers were dried over anhydrous Na_2SO_4 and concentrated under reduced pressure. The crude product was purified by flash column chromatography on silica gel using DCM/MeOH (99:1) as the eluent, affording compound 5 as a brown solid (94 mg, 0.20 mmol, yield: 63%). 1H NMR (400 MHz, (CD3)2CO) δ 8.50 (d, J = 8.8 Hz, 1H), 7.77 (d, J = 8.0 Hz, 2H), 7.44 (d, J = 8.0 Hz, 2H), 6.49 (d, J = 8.8 Hz, 1H), 4.19–4.11 (m, 2H), 3.84–3.80 (m, 4H), 3.71–3.66 (m, 2H), 3.64 – 3.60 (m, 2H), 3.60–3.53 (m, 2H), 2.86 (s, 1H), 2.42 (s, 3H).13 C NMR (101 MHz, (CD3)2CO) δ 145.78, 145.45, 145.08, 137.77, 134.22, 130.77, 128.63, 123.50, 99.90, 71.24, 71.07, 70.61, 69.34, 44.68, 21.49. ESI-MS (ion trap): m/z 467 [M + H]+. Synthesis of N-(2-(2-(2-iodoethoxy)ethoxy)ethyl)-7-nitrobenzo[c][1,2,5]oxadiazol-4-amine (6). To a solution of compound 5 (30 mg, 0.064 mmol, 1.0 equiv) in acetone (0.8 mL) was added sodium iodide (NaI, 39 mg, 0.257 mmol, 4.0 equiv). The reaction mixture was stirred at 40 °C for 16 h in a sealed vial. After completion, the mixture was diluted with ethyl acetate (EtOAc, 40 mL) and washed with brine (3 × 10 mL). The organic layer was dried over anhydrous Na_2SO_4, and concentrated under reduced pressure. The crude product was purified by flash column chromatography on silica gel using EtOAc/petroleum ether (1:1) as the eluent, affording compound 6 as a brown solid (14.5 mg, 0.034 mmol, yield: 54%). 1H NMR (400 MHz, (CD3)2CO) δ 8.54 (d, J = 8.8 Hz, 1H), 8.13 (s, 1H, broad signal), 6.55 (d, J = 8.8 Hz, 1H), 3.93–3.79 (m, 4H), 3.73–3.58 (m, 6H), 3.29 (t, J = 6.5 Hz, 2H).13 C NMR (101 MHz, (CD3)2CO) δ 145.81, 145.41, 145.02, 137.67, 123.43, 99.98, 72.35, 71.08, 70.67, 69.32, 44.59, 4.26. ESI-MS (ion trap): m/z 423 [M + H]+ Synthesis of (4-(2-(2-(2-((7-nitrobenzo[c][1,2,5]oxadiazol-4-yl)amino)ethoxy)ethoxy)ethoxy)phenyl) (3-(4-(4-((7-oxo-7H-furo[3,2-g]chromen-4-yl)oxy)butoxy)phenyl) propyl)diphenylphosphonium (PAPTP-NBD) A solution of compound 6 (14.5 mg, 0.034 mmol, 1.1 equiv) in DMF (0.5 mL) was cooled to 0 °C, and PAPTP-OH (25 mg, 0.031 mmol, 1.0 equiv, synthesized as previously reported, 10.3390/ph14020129) and potassium carbonate (K_2CO_3, 4 mg, 0.031 mmol, 1.0 equiv) were added. The reaction mixture was stirred at room temperature for 16 h. After completion, the reaction was diluted with EtOAc (30 mL) and washed with 0.5 M HCl (2 × 10 mL) followed by brine (1 × 10 mL). The combined organic layers were dried over anhydrous Na_2SO_4 and concentrated under reduced pressure. The crude product was purified by preparative HPLC and lyophilized to afford PAPTP-NBD as an orange solid (9.6 mg, 0.009 mmol, yield: 29%, purity UPLC > 95%). 1H NMR (400 MHz, (CD3)2CO) δ 8.42 (s, 1H, broad signal), 8.21 (dd, J = 9.8, 0.7 Hz, 1H), 7.93–7.68 (m, 13H), 7.26 (dd, J = 2.4, 1.0 Hz, 1H), 7.22 (dd, J = 9.0, 2.6 Hz, 2H), 7.16–7.08 (m, 3H), 6.82 (d, J = 8.6 Hz, 2H), 6.55 (s, 1H, broad signal), 6.19 (d, J = 9.8 Hz, 1H), 4.67 (t, J = 5.8 Hz, 2H), 4.24–4.21 (m, 2H), 4.09 (t, J = 5.8 Hz, 2H), 3.90–3.82 (m, 6H), 3.68 (s, 4H), 3.60–3.48 (m, 2H), 2.84 (t, J = 5.8 Hz, 2H), 2.12–1.98 (m, 6H). 13 C NMR (101 MHz, (CD3)2CO) δ 165.18, 159.24, 158.66, 153.86, 150.18, 146.37, 140.10, 136.81 (d, J = 11.5 Hz), 135.87 (d, J = 3.0 Hz), 134.61 (d, J = 10.0 Hz), 133.12, 131.25 (d, J = 12.6 Hz), 130.52, 120.83, 119.97, 117.48, 117.35, 115.48, 113.33, 109.39, 108.46, 107.37, 106.47, 94.02, 73.61, 71.46, 71.36, 70.02, 69.73, 69.23, 68.23, 49.07, 36.03 (d, J = 16.9 Hz), 27.59, 26.72, 25.49 (d, J = 3.7 Hz), 22.25 (d, J = 52.6 Hz). ESI-MS (ion trap): *m/z* 363 [M]+. (**B**) Confocal microscopy images showing the fluorescent signal in untreated cells and in those treated with fluorescent PAPTP (PAPTP-fluor) in mitochondria of $CD4^{+}CD25^{-}$ Tconv cells isolated from healthy mice before and after treatment with 100 nM PAPTP-Fluor for 30 min. The scale bar is 10 µm. Control experiment for Fig. 3A. The same cells shown in this representative image are also shown in Fig. 3A at higher magnification. (**C**) Left panels: Upper panel: Downregulation of Kv1.3 in cells treated with CRIPSR/Cas9 and Kv1.3 staining in sorted cells. Yellow: CRISPR/Cas transfected, unstained. Blue: CRISPR/Cas transfected, cells positively sorted for Kv1.3. This is the fraction, which was positively sorted and then stained with FITC-anti-Kv1.3 antibodies.For sorting, cells were collected, washed with H/S, incubated with Fc receptor blocking reagent (BioLegend, #101302; 1:50 dilution) for 15 min at 4 °C, washed, and labeled with biotin-conjugated anti-Kv1.3 antibody (Alomone Labs, #APC-101B) for 30 min at 4 °C. Following a second wash, cells were incubated with streptavidin-conjugated microbeads (Miltenyi Biotec, #130-048-101) for 30 min at 4 °C. Kv1.3-positive and -negative populations were isolated using LS columns (Miltenyi Biotec, 130-042-401). For the flow cytometry, Fc-receptors were blocked with True stain (1:50 dilution, cells were collected, washed with H/S, incubated with Fc receptor blocking reagent (Clone S17011E, BioLegend, #156604) and then an aliquot of the samples was stained with a FITC-coupled anti-rabbit IgG (1:500, Jackson Immunoresearch 711-096-152) to detect the anti-Kv1.3, which was already bound to the cells. Red: CRISPR/Cas transfected, cells negatively sorted for Kv1.3. This is the fraction, which was negatively sorted and then stained exactly as the blue fraction. The staining was done on aliquots just before retransfection of the mito-Kv1.3 construct to confirm downregulation. Lower panel: Same as above, but with aliquots that were fixed for 10 min at room temperature in 1% buffered PFA, then washed and permeabilized for 8 min with 0.1% Triton X-100 at room temperature, washed again and then stained. This confirms downregulation of extra- and intracellular Kv1.3. Right upper panel: Flow cytometry of sorted Kv1.3-negative cells that were re-transfected. Blue: Control-transfected (empty vector). Aliquots were fixed for 10 min at room temperature in 1% buffered PFA, then washed and permeabilized for 8 min with 0.1% Triton X-100 at room temperature, washed again, Fc-receptors

were blocked with True stain (1:50, Biolegend, #156604) and then stained with FITC-coupled anti-Kv1.3 (Alamone, #APC-101-F). Red: EYFP-Mito-Kv1.3 transfected cells, as above. Right lower panel: Representative dot plot showing the gating strategy for the identification of EYFP-mitoKv1.3$^+$ Annexin$^+$ and EYFP-mitoKv1.3$^-$ Annexin$^+$ cells after PAPTP treatment. Kv1.3$^-$ cells were transfected with EYFP-mitoKv1.3 construct and subsequently treated with PAPTP for 48 h. Cells were then analyzed for apoptosis using flow cytometry. (**D**) Representative images of longitudinal spinal-cord sections stained with luxol fast blue from mice receiving MOG_{35-55} activated untreated or 1 µM PAPTP-treated lymphocytes. Scale bar, 100 µm. Demyelinated are indicated with white arrows. Right: Average ± SEM of myelin area per field ($n = 3$ for mice receiving untreated lymphocytes, $n = 5$ for mice receiving 1 µM PAPTP-treated lymphocytes). *p*-value of Student's t test. (**E**) Representative images of longitudinal spinal-cord sections stained with Bielschowsky staining from mice receiving MOG_{35-55} activated untreated or 1 µM PAPTP-treated lymphocytes. Scale bar, 200 µm. Right upper panel: enlarged image from the lower EAE AT untreated sample shown on the left. Right lower panel: enlarged image from the upper EAE AT + PAPTP sample shown on the left. Please note damaged axon fibers in the EAE. (**F**) Quantification of axon damage from longitudinal sections of Blieschowsky-stained spinal cords from EAE AT and EAE AT + PAPTP animals. Quantification was performed following the method used for Fig. 4D (left panel) and according to (Theotokis et al, 2022). (**G**) Representative images of a brain section of a wild-type mouse injected with MOG_{35-55} activated untreated lymphocytes, stained with the indicated antibodies. The sections are from the same experiment shown in Fig. 3J. (**H**) Average ± SEM of the number of GFAP$^+$ cells per field in brain slices of healthy animals (Ctrl) and mice receiving untreated or 1 µM PAPTP-treated lymphocytes ($n = 3$ for each group). On the right, representative immunohistochemical images of GFAP$^+$ in brain slices from mice of the indicated groups. The images were taken from the same region for each animal. The scale bar corresponds to 100 µm. *p*-value from one-way ANOVA. (**I**) Additional examples of Klüver-Barrera dual staining performed as in Fig. 4C. Red arrows indicate infiltrated/demyelinated zones. Please note also vacuolation, as e.g., in (Morales et al, 2006) in the enlarged image.

 © The Author(s)

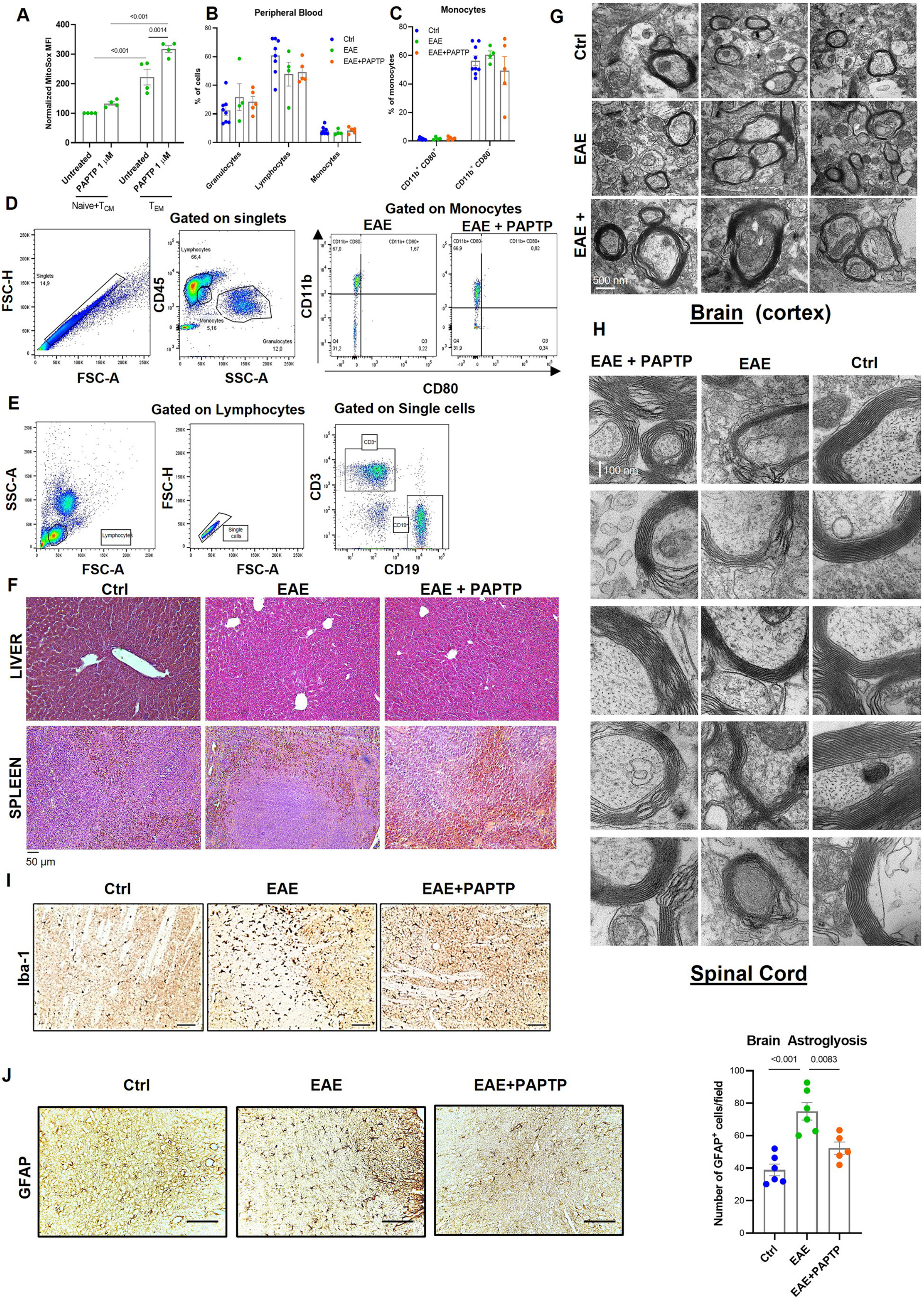
A
Normalized MitoSox MFI
<0.001
0.0014
<0.001
Untreated
PAPTP 1 μM
Untreated
PAPTP 1 μM
Naive+TCM
TEM
B
Peripheral Blood
% of cells
Ctrl
EAE
EAE+PAPTP
Granulocytes
Lymphocytes
Monocytes
C
Monocytes
% of monocytes
Ctrl
EAE
EAE+PAPTP
CD11b+ CD80+
CD11b+ CD80-
D
Gated on singlets
Gated on Monocytes
EAE
EAE + PAPTP
FSC-H
FSC-A
Singlets
14,9
CD45
SSC-A
Lymphocytes
66,4
Monocytes
5,16
Granulocytes
12,0
CD11b
CD80
E
Gated on Lymphocytes
Gated on Single cells
SSC-A
FSC-A
Lymphocytes
FSC-H
Single cells
CD3
CD19
F
Ctrl
EAE
EAE + PAPTP
LIVER
SPLEEN
50 μm
G
Ctrl
EAE
EAE + PAPTP
500 nm
Brain (cortex)
H
EAE + PAPTP
EAE
Ctrl
100 nm
Spinal Cord
I
Ctrl
EAE
EAE+PAPTP
Iba-1
J
Ctrl
EAE
EAE+PAPTP
GFAP
Brain Astrogliosis
<0.001
0.0083
Number of GFAP+ cells/field
Ctrl
EAE
EAE+PAPTP

Figure EV3. Effects of PAPTP in EAE model.

(**A**) Mean Fluorescence intensity of MitoSox in $CD4^+$ $CD62L^+$ (Naive+T_{CM}) and $CD4^+$ $CD62L^-$ (T_{EM}) lymphocytes in peripheral blood from EAE mice before (Untreated) and after 30 min treatment with 1 µM PAPTP. For each animal, data were normalized on the Mitosox Mean Fluorescence Intensity of $CD4^+$ $CD62L^+$ untreated cells. Data represent average ± SEM ($n = 4$). p-values of two-way ANOVA are indicated. (**B**) Percentages of lymphocytes, monocytes, and granulocytes in peripheral blood of mice of the indicated group at the endpoint of the experiment, evaluated using flow cytometry. Populations were gated on total single cells. Data represent average ± SEM ($n = 8$ for controls; $n = 4$ for EAE group and $n = 5$ for EAE + PAPTP group). (**C**) Percentages of $CD11b^+$ $CD80^+$ and $CD11b^+$ $CD80^-$ monocytes in peripheral blood of mice of the indicated group at the experimental endpoint. Cells were gated on $C11b^+$ monocytes. Data represent average ± SEM ($n = 9$ for controls; $n = 4$ for EAE group and $n = 5$ for EAE + PAPTP group). (**D**) Gating Strategy for identification of monocytes, lymphocytes and granulocytes (top). Representative dot plot showing $CD11b^+$ $CD80^+$ and $CD11b^+$ $CD80^-$ monocytes in peripheral blood of mice of the indicated group at the experimental endpoint (bottom). (**E**) Gating Strategy for identification of $CD3^+$ and $CD19^+$ lymphocytes in peripheral blood of mice. (**F**) Representative H&E images of livers and spleens of mice of the indicated groups. Scale bar indicated in the figure. Representative Transmission Electron Microscopy images showing neuronal (**G**) and spinal cords (**H**) myelination of mice from the indicated groups at the experimental endpoint. Scale bar indicated in the figure. (**I**) Representative immunohistochemical images of Iba-1^+ cells in brain slices from mice of the indicated group, sacrificed at 30 dpi. The scale bar corresponds to 100 µm. See enlarged images on Fig. 5K. (**J**) Average ± SEM of the number of $GFAP^+$ cells per field in brain slices of mice of the indicated groups ($n = 6$ for Ctrl and EAE, $n = 5$ for EAE + PAPTP groups). One outlier, defined with GraphPad was removed from the EAE + PAPTP group. At least 5 sections per animal were analyzed. On the right, representative immunohistochemical images of $GFAP^+$ in brain slices from mice of the indicated groups. The images were taken from the same region for each animal. The scale bar corresponds to 100 µm. (**G–J**) p-values of one-way ANOVA test are shown.

 © The Author(s)

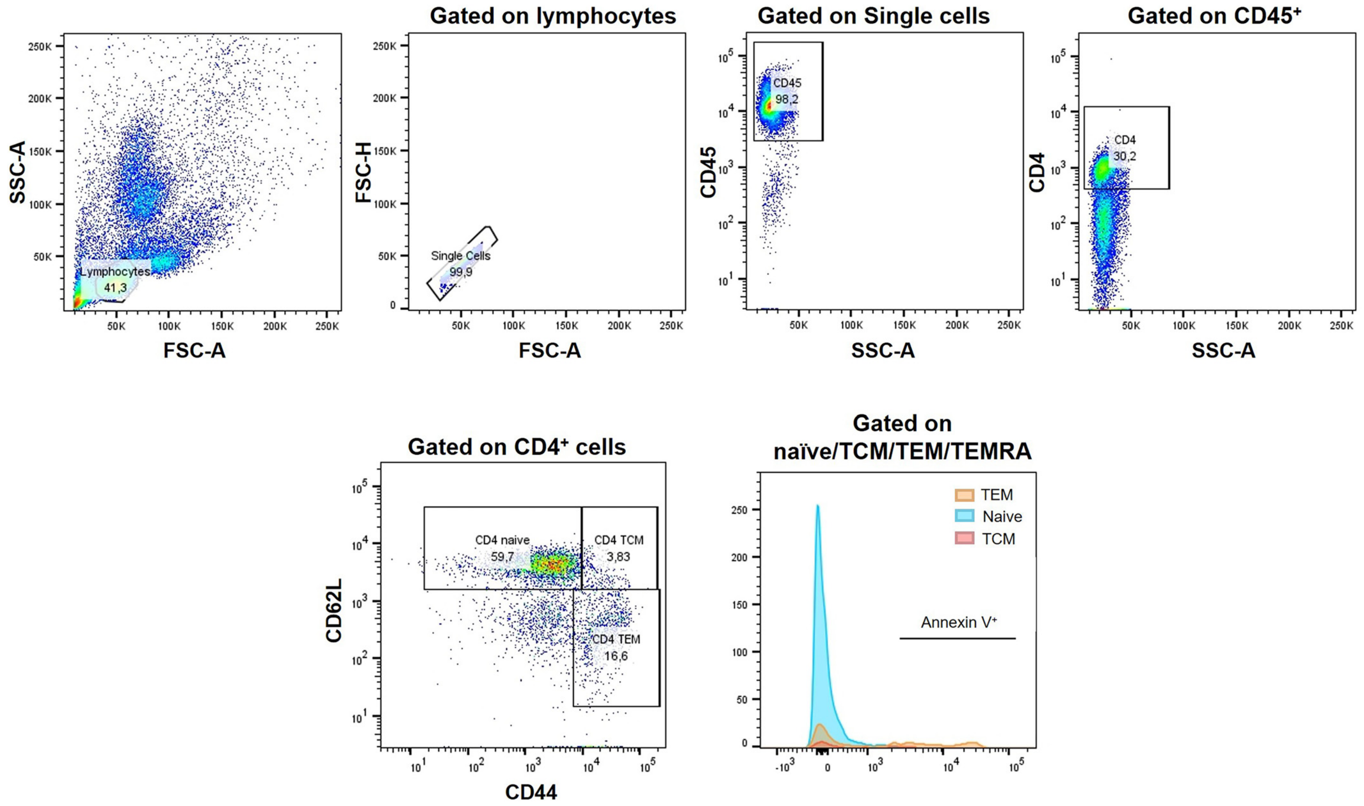

Figure EV4. Gating strategy in EAE mice blood.

Gating strategy used for the evaluation of apoptosis in naive, T_{EM} and T_{CM} lymphocytes in peripheral blood of mice.
